# Viral Oncogene–Induced DNA Damage Response Is Activated in Kaposi Sarcoma Tumorigenesis

**DOI:** 10.1371/journal.ppat.0030140

**Published:** 2007-09-28

**Authors:** Sonja Koopal, Johanna H Furuhjelm, Annika Järviluoma, Sari Jäämaa, Pawan Pyakurel, Christel Pussinen, Maria Wirzenius, Peter Biberfeld, Kari Alitalo, Marikki Laiho, Päivi M Ojala

**Affiliations:** 1 Genome-Scale Biology Program and Institute of Biomedicine, Biomedicum Helsinki, University of Helsinki, Finland; 2 Molecular Cancer Biology Program, Haartman Institute, Biomedicum Helsinki, University of Helsinki, Finland; 3 Department of Pathology and Oncology, Karolinska Institute/Hospital, Stockholm, Sweden; University of California San Francisco, United States of America

## Abstract

Kaposi sarcoma is a tumor consisting of Kaposi sarcoma herpesvirus (KSHV)–infected tumor cells that express endothelial cell (EC) markers and viral genes like *v-cyclin, vFLIP,* and *LANA.* Despite a strong link between KSHV infection and certain neoplasms, de novo virus infection of human primary cells does not readily lead to cellular transformation. We have studied the consequences of expression of *v-cyclin* in primary and immortalized human dermal microvascular ECs. We show that *v-cyclin,* which is a homolog of cellular *D-type cyclins,* induces replicative stress in ECs, which leads to senescence and activation of the DNA damage response. We find that antiproliferative checkpoints are activated upon KSHV infection of ECs, and in early-stage but not late-stage lesions of clinical Kaposi sarcoma specimens. These are some of the first results suggesting that DNA damage checkpoint response also functions as an anticancer barrier in virally induced cancers.

## Author Summary

Recent findings have indicated that DNA hyper-replication triggered by oncogenes can induce cellular senescence, which together with the oncogene-induced DNA damage checkpoint confers a barrier to tumorigenesis. Kaposi sarcoma herpesvirus (KSHV) can infect human dermal microvascular endothelial cells (ECs) in vitro, but KSHV infection does not seem to provide growth advantage to the cells, but rather leads to retarded growth. Moreover, the proliferative index has long been known to be low in KSHV-infected spindle cells in Kaposi sarcoma (KS) tumors. Our results provide an explanation for these observations by showing that activation of the DNA damage response, exerted by KSHV and a latent viral protein v-cyclin, functions as a barrier against transformation of KSHV-infected cells. Interestingly, the antiproliferative checkpoints are activated during the initial stages of KSHV infection and KS tumorigenesis. During the course of infection, the infected cells are imposed to overcome the checkpoint, and oncogenic stress elicited by the expression of v-cyclin may further contribute to the induction of genomic instability and malignant transformation.

## Introduction

Recent findings suggest that DNA damage checkpoints become activated in early stages of human tumorigenesis, leading to growth arrest or apoptosis and thereby hindering tumor progression. Likewise, very recent reports have indicated that oncogene-induced senescence triggered by DNA replication stress also has a role as a tumorigenesis barrier. DNA damage checkpoint markers like phosphorylated ATM and Chk2 kinases and phosphorylated histone H2AX and p53 are activated in precancerous lesions (early stages of tumorigenesis) of several different human cancers, including bladder, breast, colon, and lung cancer [1,2]. These checkpoint responses precede p53 mutations and the appearance of gross chromosomal abnormalities. The tumorigenic events early in the progression of major human cancer types activate the ATR/ATM-regulated checkpoint as a guard against tumor progression and genetic instability. Candidate inducers of the response include oncogenes such as *Myc* [3,4], *Ras* [5], *Cdc6* [1], *Cdc25A, E2F1,* or overexpressed *cyclin E* [6].

Kaposi sarcoma herpesvirus (KSHV, or human herpesvirus 8 [HHV8]) is a γ-2 herpesvirus implicated in all subtypes of Kaposi sarcoma (KS), in multicentric Castleman disease, and in primary effusion lymphoma (PEL) [7–9]. KSHV establishes a latent infection in host cells, where only a subset of viral genes is expressed, while viral replication is not activated [10]. KS is an angiogenic tumor that consists of proliferating infected cells that form irregular microvascular channels and extravasated infiltrating inflammatory cells. Tumor cells in KS lesions are characterized by spindle-like morphology, and they express endothelial cell (EC) markers but also have features of other cell lineages, like macrophages and smooth muscle cells [11–14]. In KS lesions, all tumor cells are latently infected by KSHV and express latent genes, such as the viral cyclin (*v-cyclin*)*,* the viral FLICE inhibitory protein (*vFLIP*)*,* and the latency associated nuclear antigen (*LANA*)*.* These latent proteins are known to impinge in the regulation of the cell cycle, cell survival, and the major tumor suppressor pathways p53 and pRb, which suggests that they are important for viral pathogenesis (reviewed in [15]).

v-cyclin is structurally similar to cellular D-type cyclin and forms an active kinase complex with cellular CDK6. v-cyclin also associates with CDK4 and CDK2, but the binding does not lead to significant activation of these kinases. As its cellular counterpart, v-cyclin–CDK6 also phosphorylates pRb and induces accelerated S-phase entry in cultured cells, but it has a remarkably broader substrate range than the cellular D-type cyclin–CDK4/6 complexes (reviewed in [16–18]). v-cyclin–CDK6 is resistant to inhibition by CDK inhibitors. Interestingly, both p27Kip1 and p21Cip1 are phosphorylated by v-cyclin–CDK6, which leads to their inactivation [19–22]. These properties of v-cyclin suggest that it can function as an oncogene. However, v-cyclin expression in primary cells was shown to induce not only DNA synthesis but also a p53-dependent growth arrest and cytokinesis defects. These growth-restricting steps were overcome by the loss of p53, which exposed the oncogenic potential of v-cyclin [23].

Previous reports have demonstrated that ECs are susceptible to both latent and productive infection by KSHV, and therefore represent a good model system for studies of the pathogenesis of this endothelial neoplasm [11,24–27]. However, the effect of KSHV infection on the growth properties and tumorigenic conversion of infected cells has remained obscure. In this study, we have assessed the consequences of v-cyclin expression and de novo KSHV infection in human ECs. Our results show that expression of v-cyclin in ECs induces senescence and strong DNA damage response, leading to centrosome amplification and growth arrest. Moreover, we have analysed the major tumor suppressor pathways upon de novo KSHV infection of ECs, and our data indicate that antiproliferative checkpoints are activated during the initial stages of KSHV infection. To determine whether DNA damage response is also associated with the development of KS, we examined early and late KS lesions for expression of the checkpoint markers.

## Results

### v-Cyclin Expression Triggers an Intra–S-Phase Arrest in Human ECs

**Figure 1:**
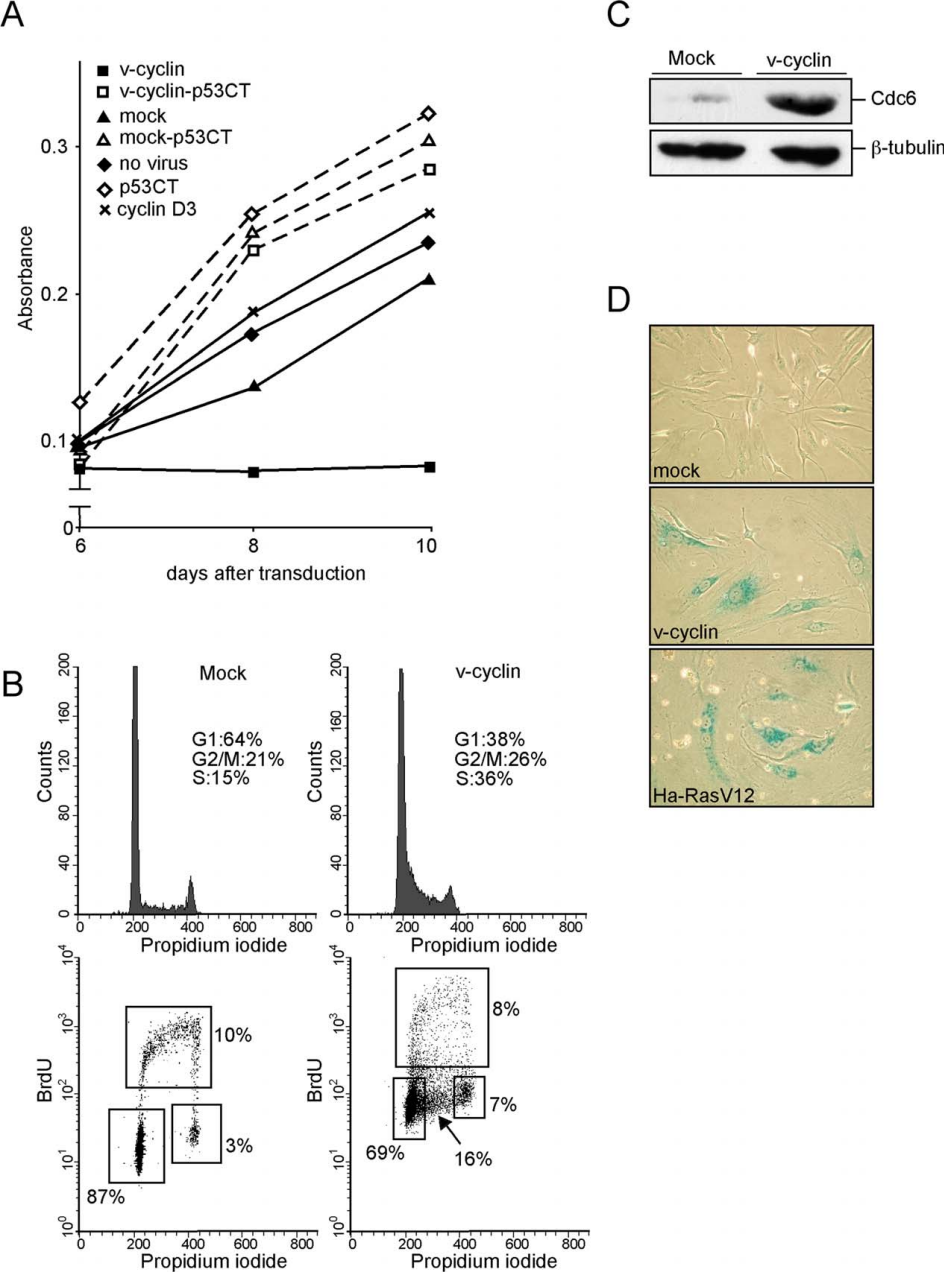
v-Cyclin Expression in ECs Triggers an Intra–S-Phase Growth Arrest (A**)** hT-HDMECs expressing mock, v-cyclin, or cyclin D3, and hT-HDMECs-p53CTer expressing mock (mock-p53CT) or v-cyclin (v-cyclin-p53CT) were assayed for metabolic activity by a MTT assay at 6, 8, and 10 d after transduction. Nontransduced hT-HDMECs (no virus) were included as a control in the assay. (B) hT-HDMECs expressing mock or v-cyclin were subjected to cell-cycle analysis by BrdU incorporation and flow cytometry. The percentages of cells at specific cell-cycle phases are indicated. The numbers represent mean values of four independent experiments. (C) Whole-cell extracts of hT-HDMECs expressing mock or v-cyclin were resolved by SDS-PAGE (12%) and immunoblotted with antibodies against Cdc6. Anti–β-tubulin was used as a loading control. (D) hT-HDMECs constitutively expressing mock, v-cyclin, or H-RasV12 were grown for 7 d. Cells were fixed and stained for senescence-associated beta-galactosidase activity.

To analyse the consequences of v-cyclin expression in a cell type naturally infected by KSHV, we used both primary human dermal microvascular ECs (HDMECs) and their telomerase (hTERT)–immortalized derivatives (hT-HDMECs). The cells were transduced with retroviruses expressing Flag-tagged v-cyclin protein (see Text S1 for the vectors used). To explore the proliferation capacity of v-cyclin–expressing hT-HDMECs (v-cyclin–ECs), freshly transduced cells were grown for 48 h and then subjected to a 3-d selection in puromycin, after which proliferation was analysed by an MTT assay. As depicted in Figure 1A, cells transduced with the control virus (vector only; mock) and nontransduced cells proliferated during the follow-up period, while the v-cyclin–ECs did not. The specific v-cyclin–associated kinase activity in these cells was comparable to v-cyclin kinase activity in PEL cells, which are naturally infected, KSHV-positive cells expressing v-cyclin (unpublished data). However, during 2-wk culture of the v-cyclin–ECs, v-cyclin protein was lost from the cells (as analysed by indirect immunofluorescence and Western blotting), and the cells started to proliferate again (unpublished data). For comparison, ECs transduced with a retrovirus expressing a cellular homolog to v-cyclin, cyclin D3, had full proliferative capacity comparable to that of the controls (Figure 1A). Thus the proliferation arrest in the v-cyclin–ECs was a specific property of this viral cyclin and not just a consequence of cyclin overexpression. To examine whether the v-cyclin–induced growth arrest in ECs was p53- dependent, we transduced hT-HDMECs constitutively expressing a dominant-negative p53 (*p53CTer;* encoding the C-terminal amino acids 302–390 of murine p53). These hT-HDMECs–p53CTer were transduced with the v-cyclin or control retrovirus. Expression of p53CTer in the ECs disturbs the wild-type (wt) p53 functions, which was observed as a growth advantage over the parental hT-HDMECs (Figure 1A). Indeed, cells coexpressing v-cyclin and p53CTer had full proliferative capacity, suggesting that the growth arrest was p53-dependent (Figure 1A).

To determine at which stage of the cell cycle v-cyclin–ECs are arrested, we analyzed their cell-cycle profile by flow cytometry 7 d after transduction. According to the DNA profile, a significantly higher proportion of v-cyclin–ECs were in the S-phase (36%) compared with the mock-transduced cells (15%; Figure 1B, top panels). Similar cell-cycle distributions were observed already at day 2 after transduction (unpublished data). Since the v-cyclin–ECs lacked proliferative capacity, the observed increase in the number of S-phase cells could result either from re-replication of nuclear DNA without subsequent mitosis (endoreduplication) or an intra–S-phase arrest. To determine precisely the nature of this cell-cycle arrest, we performed a pulse-chase experiment with BrdU. A 2-h pulse of BrdU (15 μM), followed by 5-h and 10-h chase periods, was analysed by multiparameter flow cytometry. By plotting BrdU incorporation versus DNA content, we were able to detect both BrdU-labeled and unlabeled cells. After the initial 2-h labeling period, 10% of the mock-transduced cells and 8% of the v-cyclin–ECs had incorporated BrdU (Figure 1B, bottom panels). However, the total fraction of cells in S-phase (both BrdU-positive and -negative) was higher in the v-cyclin–ECs (24%) than in the mock ECs (10%). After the 5-h and 10-h chase periods, the population of BrdU-positive, mock-transduced cells had shifted towards the G_2_/M-phase, while the profile of BrDU-positive cells in v-cyclin–ECs remained unchanged (unpublished data). The v-cyclin–ECs with an S-phase DNA content but negative for BrdU incorporation most likely represent cells that arrested at the S-phase before the BrdU pulse. This data indicates that the v-cyclin–ECs are not undergoing endoreduplication, but are arrested at the S-phase.

The intra–S-phase checkpoint is activated as a result of DNA replication stress [28]. Previous studies have demonstrated that v-cyclin–CDK6 interacts with and phosphorylates components of the origin recognition complex such as Orc1 and Cdc6 [29]. Recent observations have established Cdc6 as a key replication initiation protein whose stability is increased by CDK phosphorylation [30]. The accumulated Cdc6 binds to the origin recognition complex, and promotes the initiation of DNA replication [31]. To explore if v-cyclin expression in the ECs induces premature or aberrant firing of the replication origins, we analysed the protein level of Cdc6 in the v-cyclin–ECs. v-cyclin–ECs had highly elevated levels compared with the mock-ECs, demonstrating that v-cyclin expression induces accumulation of the essential licensing factor Cdc6 in ECs (Figure 1C). Similar accumulation of Cdc6 has also been seen in response to expression of other oncogenes such as Mos [1].

Oncogene-induced DNA replication stress induces senescence in human diploid cells [1,5]. To address whether a viral oncogene can also induce senescence, we stained the v-cyclin–ECs for the senescence-associated beta-galactosidase [32,33] (see Text S1). H-RasV12–expressing ECs were used as a positive control. Both the H-RasV12– and v-cyclin–ECs stained positive for senescence-associated beta-galactosidase, while the mock-transduced ECs remained negative (Figure 1D). In addition to the positive signal from the senescence marker, the v-cyclin–ECs appeared significantly larger and flatter than cells expressing the mock virus.

### v-Cyclin Induces DNA Damage Response in ECs

In response to replication stalling and DNA damage, checkpoint pathways are activated. We therefore analysed whether v-cyclin expression was evoking a DNA damage checkpoint in ECs. The occurrence of a DNA damage response can be ascertained by monitoring the appearance of specific markers such as phosphorylated histone H2AX (γ-H2AX), phosphorylation of the ATM kinase (on Ser1981; pS-ATM), or Chk2 kinase (on Thr68; pT-Chk2), focal staining of p53-binding protein 1 (53BP1), or increase in Ser15-phosphorylated p53 (pS-p53).

**Figure 2:**
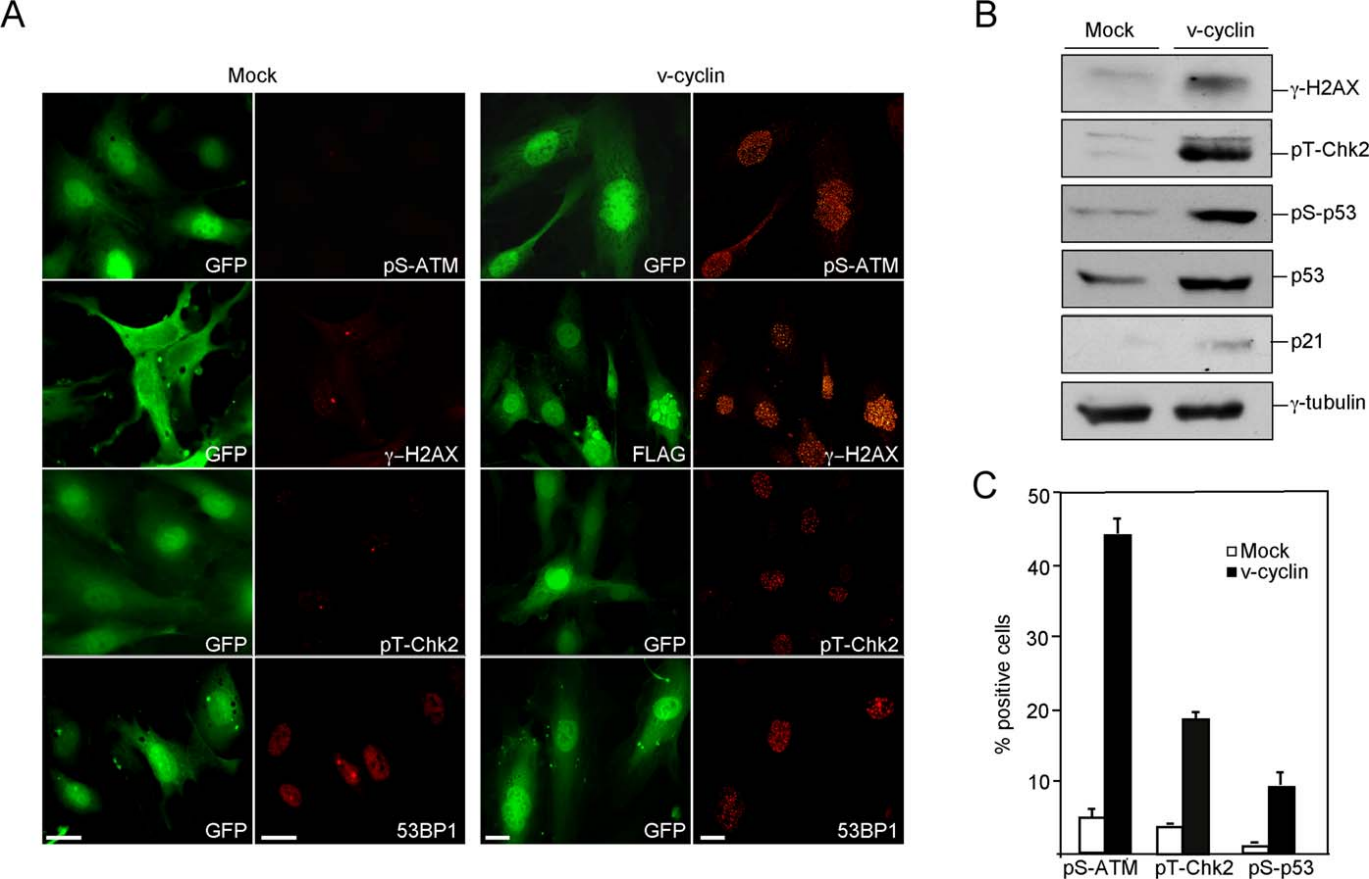
DNA Damage Response in v-Cyclin–ECs (A) hT-HDMECs expressing mock (pBMNIresEGFP) or v-cyclin (KpBMNIresEGFP) retroviruses were analysed at day 5 after transduction for expression of GFP and the indicated DNA damage markers. The labeling with anti-gamma-H2AX antibody requires EtOH fixation, which leads to a partial loss of soluble GFP and thus reduced signal. Therefore, to allow simultaneous detection of γ-H2AX together with mock or v-cyclin, the cells in these panels were labeled also with α-GFP or α-Flag (for v-cyclin) antibodies, respectively. Scale bar = 20 μm. (B) hTERT-HDMECs expressing mock or v-cyclin at day 7 were immunoblotted with antibodies indicated on the left. γ-tubulin was used as a loading control. (C) hT-HDMECs expressing mock or v-cyclin were analysed at day 5 by indirect immunofluorescence to determine the percentage of cells positive for the indicated DNA damage markers. The average of two individual experiments is shown. Approximately 200 cells were analysed per sample.

Cytological and biochemical analyses of v-cyclin–ECs (both immortalized and primary ECs) showed strong induction of all the above-mentioned DNA damage markers starting at 2–3 d after transduction (Figure 2 for hT-HDMECs and Figure S1 for the primary HDMECs). Although 53BP1 expression was detected as uniform nuclear staining in the mock-virus–transduced ECs, its signal in the v-cyclin–ECs appeared dramatically different, and localized into bright intranuclear foci (Figure 2A, bottom panel). These results suggested that v-cyclin activates the ATM–Chk2 pathway in ECs. In accordance with previous studies [23,34], the v-cyclin–ECs also showed p53 stabilization and p21 induction (Figure 2B). In contrast, little or no signal for the DNA damage markers was detected in ECs transduced with the mock-virus (Figure 2A and 2B) or with retrovirus expressing cyclin D3 (unpublished data), consistent with the absence of a DNA damage response. Quantitation of the number of cells expressing markers for activated DNA damage response confirmed a significant increase in their expression in the v-cyclin–ECs compared with the mock-transduced ECs (Figure 2C). The pronounced induction of γ-H2AX and the S-phase promoting capacity (Figure 1B and 1C) suggested that the DNA damage checkpoint induced by v-cyclin expression was provoked by DNA replication stress in the ECs.

### Centrosome Amplification and Multinucleation in v-Cyclin–ECs

**Figure 3:**
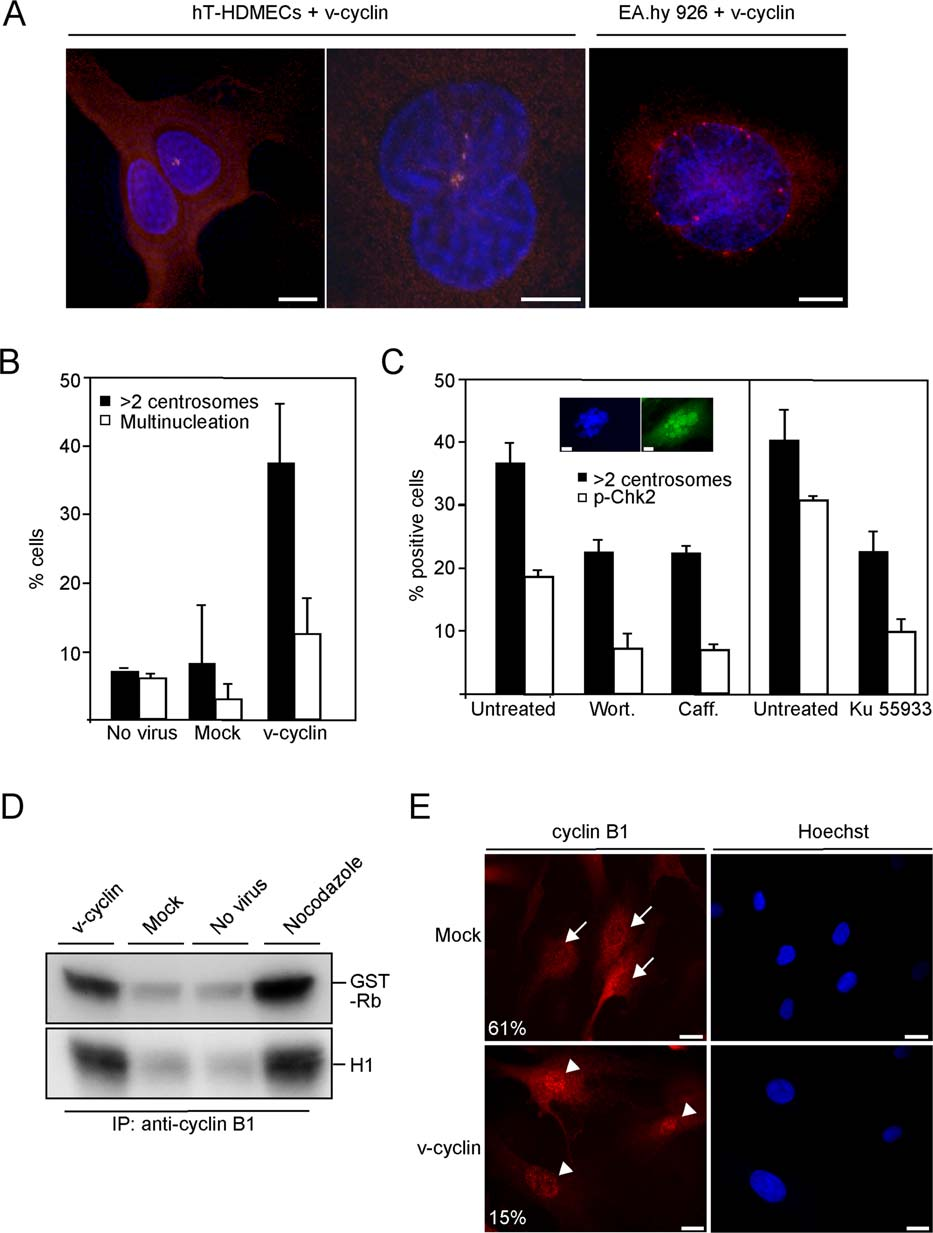
Centrosome Amplification and Multinucleation in the v-Cyclin–ECs (A) hT-HDMECs (left and middle panels) or EA.hy 926 cells (right panel) expressing v-cyclin were analysed for centrosomes at day 3 or 5, respectively, by anti–γ-tubulin antibody (red) and DNA by Hoechst staining (blue). Scale bar = 10 μm. (B) Quantitation of the number of centrosomes and multinuclear cells in the nontransduced (No virus), mock, or v-cyclin–expressing hT-HDMECs at day 3 after transduction. The average of two independent experiments and analysis of about 200 cells per sample is shown. (C) Inhibition of the ATM-Chk2–dependent checkpoint decreases centrosome aberrations in v-cyclin–ECs. v-cyclin–ECs were treated with wortmannin (200 nM) or caffeine (2 mM) 3 d after transduction for 4 h (left graph), or with KU-55933 (2 μM) 6 d after transduction for 3 d (right graph). Treated and untreated cells were analyzed to determine the percentage of cells with activated Chk2 (by anti–pT-Chk2) or with more than two centrosomes (by anti–γ-tubulin). The average of two independent experiments is shown. More than 200 cells were analyzed per sample. Insert: inhibition of the ATM-Chk2–dependent checkpoint in v-cyclin–ECs leads to formation of multinucleated syncytia-like cells. Scale bar = 10 μm. (D) Cell extracts of nontransduced hT-HDMECs (No virus), or hT-HDMECs expressing mock or v-cyclin for 2 d were immunoprecipitated with anti–cyclin B1 antibodies, and subjected to in vitro kinase assay using GST-Rb and histone H1 as substrates. hTERT-HDMECs treated with nocodazole (75 ng/ml) were used as a positive control. Kinase activity was determined by autoradiography after SDS-PAGE (12%). (E) hT-HDMECs expressing mock or v-cyclin were labeled with α-cyclin B1 (red) and Hoechst (blue) at day 6 after transduction. Slides were microscopically analyzed to determine the percentage of cells where cyclin B1 was excluded from the nucleus (% on the left). About 200 cells were counted per sample. Arrows indicate cells with cyclin B1 signal excluded from the nucleus, and arrowheads indicate the cells with nuclear cyclin B1 signal. Scale bar = 20 μm.

Expression of v-cyclin in mouse embryonic fibroblasts (MEFs) has been shown to induce centrosome amplification [23], which prompted us to investigate whether the v-cyclin–ECs also displayed supernumerary centrosomes. We analysed the number of centrosomes in v-cyclin–expressing primary HDMECs, hTERT-HDMECs, or endothelial EA.hy926 cells by indirect immuofluorescence. Anti–γ-tubulin labeling of the cells revealed centrosomal amplification either as clustered centrosomes in HDMECs and hT-HDMECs (Figure 3A, left and middle panels) or as multiple centrosomes surrounding the nucleus in EA.hy926 cells (Figure 3A, right panel). Amplification of centrosomes was often accompanied with bi- or multinucleation. Quantitation of these results depicted in Figure 3B for the hT-HDMECs shows a significant increase (4.2-fold) in the proportion of the v-cyclin–ECs with more than two centrosomes.

To investigate whether abrogation of the ATM-Chk2–dependent checkpoint would facilitate proliferation of the v-cyclin–ECs, we treated the cells with caffeine and wortmannin to inhibit ATM and ATR kinases (Figure 3C, left graph), or with a specific ATM inhibitor KU-55933 (Figure 3C, right graph). The inhibition was analysed by immunofluorescence analysis, which showed reduced phosphorylation of Chk2 as well as reduction of focal staining of 53BP1 in the KU-55933–treated cells. Treatment (unpublished data) with all of the inhibitors partially rescued the centrosomal aberrations in the v-cyclin–ECs (Figure 3C), but did not lead to an increase in the net cell numbers (unpublished data). However, inhibition of ATM–Chk2 by the chemical inhibitors led to an increase in multinucleated syncytia-like cells (Figure 3C, insert), suggesting that the S-phase arrest was released, but this resulted in aberrant mitosis and subsequent “mitotic catastrophe.”

In order to explore if the centrosome amplification in v-cyclin–ECs was dependent on p53, we analysed the centrosome numbers in hT-HDMECs constitutively expressing the dominant-negative p53CTer together with v-cyclin, and compared them with the v-cyclin–ECs. Inactivation of p53 by the C-terminal mutant led to a 40% decrease in the cells with abnormal centrosome numbers (unpublished data), suggesting that the amplification of centrosomes by v-cyclin is p53-dependent.

About one-third of the cells harboring more than two centrosomes were either binucleated (as in Figure 3A), multinucleated, or had micronuclei, indicating that karyokinesis (nuclear division) had occurred without concomitant cytokinesis. Yet, the proportion of cells undergoing mitosis in the v-cyclin–ECs was very low as detected by an antibody against serine 10–phosphorylated histone H3 (unpublished data). Interestingly, the v-cyclin–ECs showed increased cyclin B1–associated kinase activity when compared with the mock-transduced cells as well as an increase in the nuclear localization of cyclin B1 (Figure 3D and 3E). This suggests that the cells were unable to switch off the cyclin B1 activity, which in turn can lead the cells to aberrant mitosis (karyokinesis).

### v-Cyclin–Induced DNA Damage and Centrosome Amplification Are Dependent on CDK6

**Figure 4:**
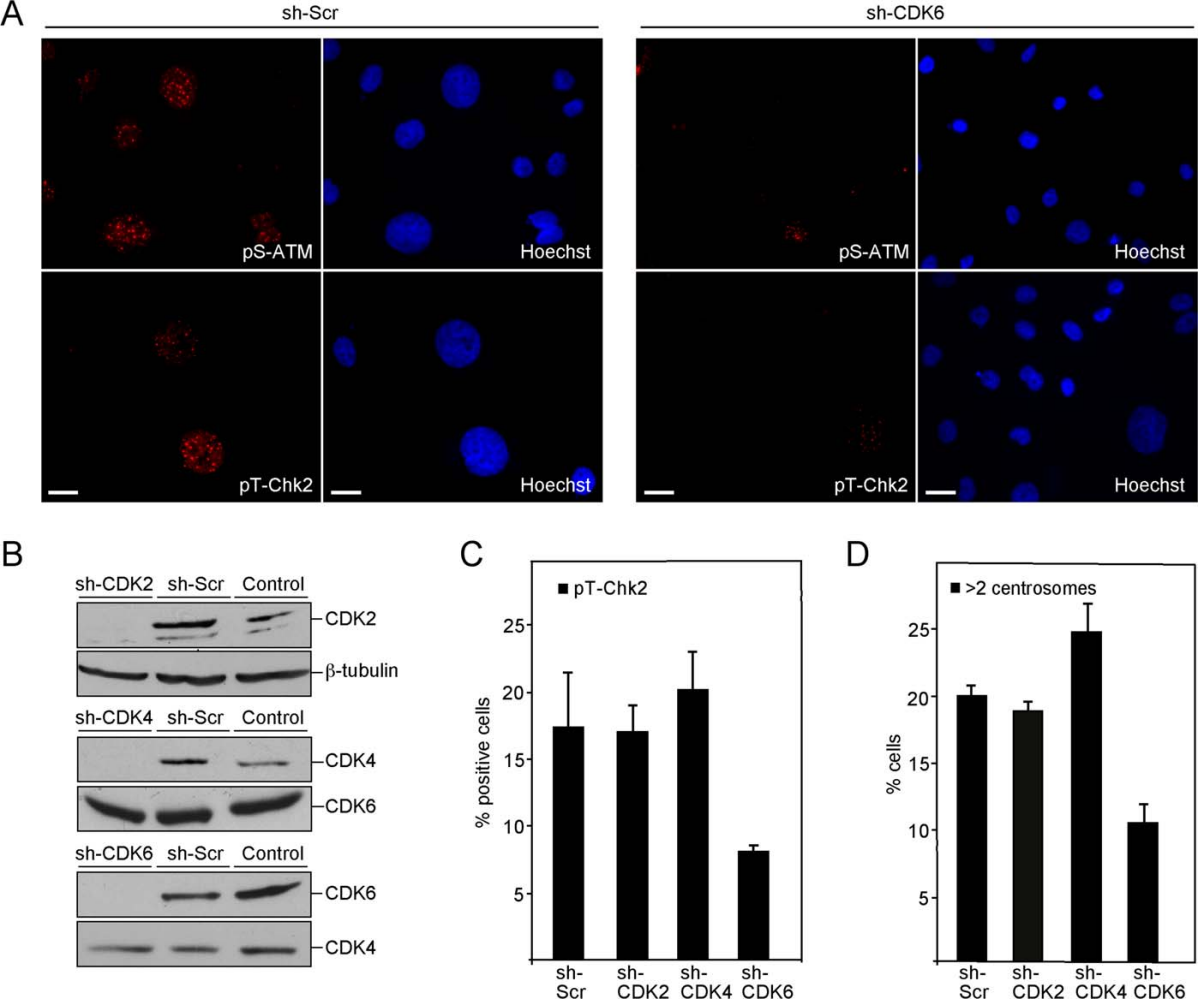
v-Cyclin–Induced DNA Damage Response and Centrosome Amplification Are CDK6-Dependent (A) shRNA-encoding EA.hy926 cells sh-Scramble or sh-CDK6 were transduced with v-cyclin retrovirus, and grown for 5 d. Transduced cells were labeled with antibodies against pS-ATM, pT-Chk2, and Hoechst (blue) for DNA. Scale bar = 20 μm. (B) Depletion of CDK2, CDK4, or CDK6 expression in EA.hy926 cells. The cells were stably transduced with the shRNA-encoding lentiviruses targeting either CDK2, CDK4, CDK6, or a random noncoding sequence (sh-Scr). Whole-cell extracts were resolved by SDS-PAGE (12%) and immunoblotted with antibodies against CDK2, CDK4, CDK6, and β-tubulin. (C) shRNA-encoding EA.hy926 cells sh-CDK2, sh-CDK4, sh-CDK6, or sh-Scr were transduced with v-cyclin retrovirus as in (A), and labeled with antibody to pT-Chk2 to determine the percentage of cells with active Chk2. The average of two independent experiments with at least 200 cells analysed per sample is shown. (D) The sh-CDK2, sh-CDK4, sh-CDK6, or sh-Scr cells were transduced with v-cyclin retrovirus as in (A), and labeled with anti–γ-tubulin antibodies and Hoechst to analyse the centrosome numbers. The average of two independent experiments with at least 200 cells analysed per sample is shown.

Next, we sought to determine whether CDK activity is required for the observed DNA damage response and centrosome amplification in v-cyclin–ECs. To this end, we depleted CDK2, CDK4, or CDK6 expression in EA.hy926 cells using lentivirus-mediated RNA interference. EA.hy926 cells stably expressing small hairpin RNA (shRNA) specific for the above-mentioned kinases or control shRNA (Scramble) were first subjected to immunoblotting, which indicated very efficient downregulation (90%–95%) of the target proteins. β-tubulin was used as a loading control for the CDK2 immunoblots (Figure 4). Silencing specificity for CDK4 and CDK6 was controlled by reciprocal immunoblotting (i.e., CDK6 for CDK4 shRNA-expressing cells and vice versa; Figure 4B).

The CDK-downregulated cells were transduced with the v-cyclin–expressing retrovirus, and analyzed 5 d after transduction for DNA damage markers and centrosome numbers by indirect immunofluorescence. Cytological analysis using antibodies against pS-ATM and pT-Chk2 indicated that the DNA damage response was readily activated in the control EA.hy926 cells upon expression of v-cyclin (Figure 4A, left panels). Depletion of CDK6 resulted in a significant reduction of phosphorylation on ATM and Chk2, indicating that v-cyclin–induced DNA damage checkpoint was dependent on CDK6 expression (Figure 4A and 4C). This is in accordance with previous studies demonstrating that CDK6 is the in vivo catalytic subunit of v-cyclin in the patient-derived PEL cells [35]. Accordingly, CDK6 expression was also required for the v-cyclin–induced centrosome amplification (Figure 4D). In contrast, depletion of CDK4 or CDK2 did not decrease the DNA damage response or centrosome numbers, indicating that v-cyclin–induced DNA damage and centrosome amplification are not dependent on either of these cyclin-dependent kinases (Figure 4C and 4D). Interestingly, after CDK6 knockdown, the morphology of most of the v-cyclin–ECs returns to normal, i.e., the nuclei are of normal size and the cells do not display a flattened phenotype (Figure 4A, shCDK6 panels).

### Antiproliferative Checkpoints Are Activated in KSHV-Infected ECs

**Figure 5:**
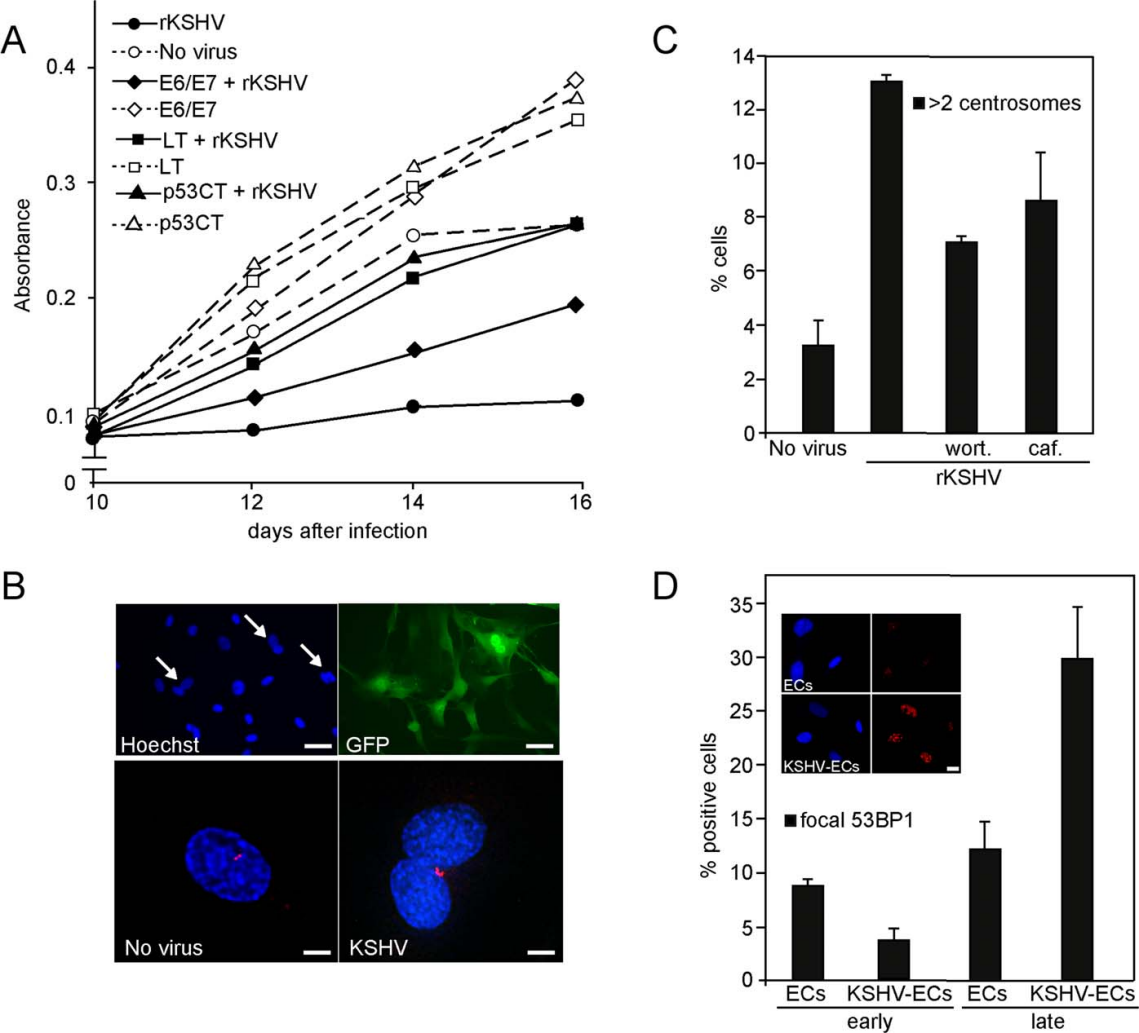
Antiproliferative Checkpoints Are Activated in KSHV-Infected ECs (A) hT-HDMECs, hT-HDMEC–p53CTer, hT-HDMEC–LT, and hT-HDMEC–E6/E7 were infected with rKSHV.219 virus and grown for 9 d. Proliferation of these cells in relation to noninfected cells was determined by the MTT assay at 10, 12, 14, and 16 d after infection. (B) Top panels: KSHV-ECs grown for 7 d and stained with Hoechst. GFP is expressed from the recombinant virus. Arrows indicate binucleated cells. Scale bar = 50 μm. Lower panels: Noninfected (No virus) and KSHV-ECs were analysed for centrosomes by anti–γ-tubulin antibodies (red) and DNA by Hoechst staining (blue) at day 7. Scale bar = 5 μm. (C) KSHV-ECs were treated with wortmannin (200 nM) or caffeine (4 mM) for 24 h, and analysed for the centrosome numbers by γ-tubulin antibodies. Untreated as well and noninfected (No virus) ECs were used as controls. Percentage of cells with aberrant centrosome numbers is shown as an average of two independent experiments and analysis of at least 200 cells per sample. (D) KSHV-ECs grown for 2 wk (early) or approximately 10 wk (late), and their passage-matched, noninfected ECs were labeled with anti-53BP1 antibodies to address activation of the DNA damage response. The bar graph shows the percentage of cells with intranuclear 53BP1-positive foci. About 200 cells were counted per sample. The insert depicts noninfected (ECs) and late KSHV-ECs showing intranuclear 53BP1 foci in the latter one (bottom right panel). Scale bar = 20 μm.

We then addressed the effects of KSHV infection on the DNA damage response of ECs. To analyse proliferation, we generated de novo KSHV-infected ECs (KSHV-ECs) by infecting the hT-HDMECs with a recombinant KSHV expressing GFP (rKSHV.219 [36]). The establishment of KSHV latent infection was confirmed by immunofluorescence using antibodies against the latent nuclear antigen, LANA (unpublished data). Proliferation was analysed by the MTT assay. The KSHV-ECs proliferated at a very slow rate compared with the noninfected, passage-matched parental cells (Figure 5A), suggesting that growth-limiting mechanisms were activated upon KSHV infection.

To investigate potential growth-suppressive pathways, we took the hT-HDMEC–p53CTer, and also prepared hT-HDMECs stably transduced with retroviruses expressing SV40 large T antigen (hT-HDMEC-LT) or the human papillomavirus oncogenes E6 and E7 (hT-HDMEC-E6/E7) to abrogate the function of both the p53 and pRb tumor-suppressor pathways. Expression of all of these genes led to a growth advantage over the parental hT-HDMECs (Figure 5A). After infection with KSHV, all these cell lines proliferated faster than the KSHV-infected normal hT-HDMECs (Figure 5A), indicating that the growth arrest was overcome. The proliferation rate was similar in cells expressing p53CTer and those expressing the multifunctional viral oncogenes. Although the presence of hTERT in these cells may alter the experimental conditions, this is suggesting that the p53 pathway primarily restricted the proliferation of KSHV-ECs. Interestingly, the KSHV-ECs started to spontaneously grow at a faster rate approximately 3–4 wk after infection (Figure S2A). The recovery from this crisis period was accompanied by an increase in LANA signal in the KSHV-infected cells (Figure S2B).

A previous report [37] showed that KSHV infection of human umbilical vein ECs (HUVECs) induced abnormal centrosome duplication and multinucleation. We therefore sought to determine whether centrosome aberrations occurred also in our KSHV-ECs by labeling them with the γ-tubulin antibodies at 7 d after infection. This revealed that the KSHV-infected ECs had a marked increase (>4-fold) in the proportion of cells with more than two centrosomes (Figure 5B, lower right panel). Furthermore, several of the cells with amplified centrosomes were also binucleated (Figure 5B, top two panels). Centrosome aberrations may arise as a result of DNA damage-induced checkpoints, and recent data indicate the involvement of the checkpoint kinases Chk1 and Chk2 in this process (reviewed in [38,39]). To explore the involvement of the ATM–Chk2 pathway in the KSHV-induced centrosome amplification, we treated the cells with pathway inhibitors followed by analysis of the centrosome numbers. This led to a marked decrease in the population of cells with abnormal centrosome numbers (Figure 5C), suggesting the involvement of the ATM–Chk2 pathway in the centrosome amplification of KSHV-infected ECs. We did not observe the multinucleated syncytia-like cells as seen in the v-cyclin–ECs (Figure 3C, insert), suggesting that expression of the other KSHV latent genes attenuated the strong oncogenic stress elicited by v-cyclin.

Cytological analysis showed no increase in the DNA damage markers (γ-H2AX, p-ATM, pT-Chk2, 53BP1, or pS-p53) during the growth arrest phase of KSHV-infected ECs (early KSHV-ECs) compared with the parental noninfected hT-HDMECs (Figure 5D). However, after overcoming the crisis period, the proliferating KSHV-ECs (late KSHV-ECs) displayed an increased DNA damage response as indicated by the appearance of intranuclear 53BP1 foci (Figure 5D).

Nutlins are recently discovered small-molecule inhibitors that competitively bind MDM2 at the p53-binding pocket and prevent the destabilization of p53. This leads to activation of the p53 pathway [40]. Recent results suggest that the cytotoxic effect of Nutlin-3a in cancer cells is enhanced by intrinsic DNA damage signaling in the cells [41], and establish the 53BP1 protein as a critical mediator of Nutlin-3a cytotoxicity [42]. To provide further evidence of the involvement of DNA damage signaling in KSHV infection of ECs, we treated the proliferating, post-crisis KSHV-ECs and their parental noninfected cells with Nutlin-3a for 24, 48, or 96 h, and analysed them for cell viability by trypan blue exclusion. As shown in Figure S3, Nutlin-3a treatment specifically increased apoptosis of KSHV-infected ECs, but had a minimal effect on the viability of the parental noninfected cells. These data provide additional evidence that DNA damage signaling is activated in KSHV-infected ECs. We have recently shown that Nutlin-3a also dissociates p53 interaction with LANA in KSHV-infected patient-derived lymphoma cells [41], providing evidence that p53-dependent apoptosis is restrained in KSHV-infected cells, most probably by LANA-mediated binding to p53. Our results from Nutlin-3a–treated KSHV-ECs further support the fact that KSHV-infected cells cannot proliferate or survive in the presence of active p53.

### Activation of DNA Damage Response in KS Skin Lesions

**Figure 6:**
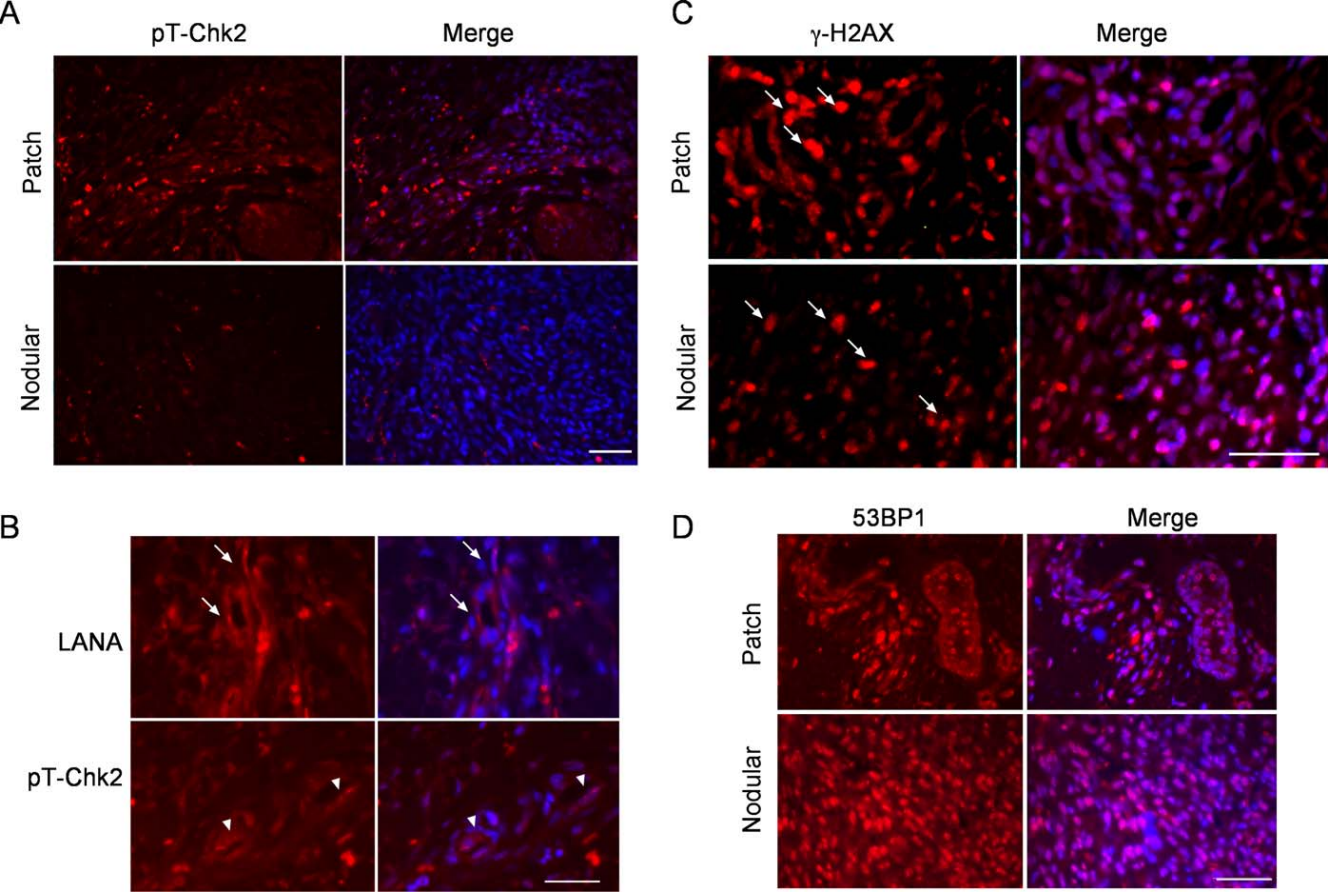
DNA Damage Response Is Activated in Early-Stage KS Lesions (A) Paraffin-embedded sections of early-stage (Patch) and late-stage (Nodular) KS skin tumors were stained for pT-Chk2, and nuclei were counterstained with Hoechst 33342. Images were captured at 40× magnification. (B) An early, patch-stage KS lesion stained for LANA and pT-Chk2, and counterstained with Hoechst 33342. Images were captured at 63× magnification, and represent consecutive sections. Arrows indicate nuclear, punctate LANA staining, and arrowheads indicate the pT-Chk2–positive nuclei also displaying a nuclear, punctate signal. (C) Early-stage (Patch) and late-stage (Nodular) KS skin lesions were stained for γ-H2AX, and nuclei were counterstained with Hoechst 33342. Arrows indicate infiltrated red blood cells**.** Images were captured at 40× magnification. (D) Early-stage (Patch) and late-stage (Nodular) KS lesions were stained for 53BP1, and nuclei were counterstained with Hoechst 33342. Images were captured at 63× magnification. Scale bars in all panels = 50 μM.

Our results suggest that KSHV infection and, more specifically, the expression of a viral latent gene can induce the activation of DNA damage signaling. To determine whether DNA damage response is activated in KS tumors as it is in several human cancers and especially in premalignant lesions [2,6], we analysed expresssion of the activated Chk2 in early-stage (patch) and late-stage (nodular) cutaneous lesions of KS (Figure 6). All KS lesions used in the study were confirmed for LANA expression by staining with anti-LANA antibodies (Figure 6B and unpublished data). The early-stage lesions are characterized by proliferating ECs forming irregular vascular spaces, associated with extravasation of red blood cells [43]. In the late nodular stage, the tumor consists of bundles of spindle cells with irregular slit-like vasculature, which is filled with erythrocytes. Immunohistochemistry for pT-Chk2 showed speckled nuclear staining in all early-stage KS skin lesions (*n* = 5). This staining could be blocked by a specific peptide (Figure S5). The signal was primarily detected in the patch-like tumor islets in the dermis (Figures 6A and S4A, top two panels), but, interestingly, it was significantly weaker for eight out of nine nodular KS cases (*n* = 9; Figures 6A and S4A, bottom two panels).

The data suggested that ATM–Chk2 signaling is activated in the early KS lesions. To expand these studies using other DNA damage markers, we examined the phosphorylation status of H2AX (γ-H2AX) and the intranuclear localization of 53BP1, both markers of the DNA double-strand break (DSB) checkpoint. Immunohistochemistry of early (*n* = 3) and late KS (*n* = 5) lesions revealed expression of γ-H2AX in both stages (Figures 6C and S4B). However, the signal intensity in the late-stage KS tumors was weaker than in the early KS tumors (Figure 6C, inserts). The infiltrated erythrocytes, typical for especially late-stage KS skin tumors, gave a very prominent autofluorescence signal, which is, however, easily separatable from the specific signal due to the absence of Hoechst staining (arrows in Figures 6C and S4B). Intriguingly, 53BP1 was mostly localized to discrete nuclear foci in the early patch KS sections (*n* = 4; Figure 6D, top panels), thus resembling the DNA damage foci in irradiated cells in culture. In two of five late-stage KS samples analyzed, 53BP1 gave a very strong but more uniform nuclear signal (Figure 6D, bottom panels) than in the early lesions. Taken together, the data suggests that DNA damage checkpoint is activated especially in the early stages of KS tumorigenesis.

## Discussion

The strong association between KSHV infection and development of KS, PEL, and multicentric Castleman disease has provided compelling evidence that KSHV is a tumorigenic virus. KSHV is well-equipped to engage important cellular signaling pathways such as cell cycle, apoptosis, angiogenesis, and immune evasion (reviewed in [15]). However, direct cellular transformation by KSHV in cell culture occurs only rarely, suggesting that additional genetic alterations are required for KSHV tumorigenesis. We show here that KSHV infection does not confer a growth advantage to endothelial cells, and that intrinsic DNA damage signaling is activated in the KSHV-ECs. This is further supported by our recent finding of activated DNA damage response in KSHV-induced lymphomas (PELs; [41]). Our results suggest that the oncogenic stress elicited by the latent viral protein v-cyclin promotes deregulated entry into the S-phase, thereby inducing DNA damage. The resulting checkpoint activation leads to growth arrest and senescence in the EC. Interestingly, overexpressed cyclin E, Cdc6, and Ras, which all share the ability to promote unscheduled S-phase entry, have recently been shown to induce cellular senescence and DNA damage response due to deregulated DNA replication [1,5,6].

Activated DNA damage signaling in KSHV-infected ECs was observed only in the proliferating “post-crisis” population of cells. It is possible that in the very early stages of de novo KSHV infection, when the cells do not grow at all, there can be other growth-restraining mechanisms related to the establishment of latent infection. These may initially affect v-cyclin function and thereby suppress the activation of the DNA damage checkpoint. Although the KSHV-ECs start to proliferate after overcoming the growth arrest, they still grow slower than noninfected cells. This is accompanied by induction of markers for the activated DNA damage checkpoint. The induction of these markers was less pronounced in KSHV-infected ECs than in the v-cyclin–ECs, which may be due to expression of other latent KSHV genes, such as *LANA,* that can interfere with the p53 and pRb tumor suppressor pathways [44,45]. However, inhibition of p53 function by LANA did not seem to be sufficient initially after infection and establishment of the latency, since an increase in the net propagation of KSHV-infected cells required abrogation of p53 signaling. Nevertheless, the increased proliferative potential of KSHV-infected ECs after the initial crisis period correlated with an increase of the LANA signal of infected cells, suggesting that p53 function was suppressed when critical levels of LANA expression were achieved. This underscores the importance of p53 in constraining KSHV pathogenesis.

It is intriguing to speculate that the accumulation of the key replication initiation protein, Cdc6, would cause untimely initiation of DNA replication and lead to formation of DNA DSBs. This is supported by the strong induction of phosphorylated ATM and Chk2 kinases in these cells along with the positive signals from the ATM substrates, γ-H2AX, and p53 phosphorylated on Ser 15 as well as focal staining of 53BP1. On the other hand, it is possible that v-cyclin deregulates cellular DNA replication or simply mimics DSBs by upregulating certain cellular factor(s) involved in the DNA damage pathway. There is growing evidence that several DNA viruses especially trigger DNA damage response in infected cells [46]. In most cases, the DNA damage response is involved in viral replication processes, and therefore the viruses have developed ways to inhibit or circumvent the host cell [47]. Human cytomegalovirus has been shown to mislocalize ATM, ATR, and Chk1 to limit the function of DNA repair proteins [48]. Interestingly, recent reports identify activated DNA damage signaling as an important mechanism both in Epstein-Barr virus lytic replication [49] and in Epstein-Barr virus oncogenesis [48].

Chk2 is normally activated as a result of DNA damage to arrest the cell cycle. It functions as an effector kinase, and prevents the activation of the cyclin B1–CDK1 complex required for the entry into mitosis. Inhibition of Chk2 and checkpoint signaling in the growth-arrested v-cyclin–ECs by treatment with chemical inhibitors induced mitotic defects (syncytia formation) and concomitant cell death (unpublished data). This together with the increased cyclin B1–CDK1 activity in v-cyclin–ECs suggest that v-cyclin was capable of eliciting also mitotic signals, but in the absence of activated Chk2, v-cyclin–induced signaling resulted in mitotic catastrophe. This is consistent with the previously identified role of Chk2 as the negative regulator of mitotic catastrophe [50].

In accordance with the previous findings by Verschuren and coworkers [23] as well as Pan and colleagues [37], we observed induction of supernumerary centrosomes and multinucleation both by v-cyclin expression and KSHV infection. These results suggest that, like other viral oncogenes (E7, E1A, and LT [51]), v-cyclin can promote chromosomal instability by disrupting the centrosome cycle. Abnormal, oncogene-induced centrosome duplication has recently been shown to require CDK2 activity [52]. The v-cyclin–induced centrosome amplification required the v-cyclin kinase partner CDK6, but not CDK2, which suggests that v-cyclin–CDK6 activity may directly drive the aberrant centrosome duplication.

Growing evidence indicates that alterations in centrosome number or function contribute to genomic instability and aneuploidy in advanced human cancers. Our results suggest that v-cyclin may contribute to the centrosomal abnormalities in KSHV-infected cells, which could lead to the induction of genomic instability and thereby predispose the cells to malignant transformation. Recently, KSHV LANA was shown to induce centrosome aberrations and increased multinucleation, indicating that other viral genes can also increase the incidence of genomic instability and promote KSHV-mediated tumorigenesis [53].

The finding of activated DNA damage signaling predominantly in the early-stage KS lesions suggests that this recently discovered anticancer barrier is also important in KS tumorigenesis. This inducible anticancer mechanism is activated in the premalignant lesion subject to “oncogenic stress” [2,6], and it imposes a selective pressure to gain mutations that compromise the checkpoint. The observed checkpoint activation in the early KS lesions correlates with their relatively low proliferation index [54] and the reduction of apoptosis in the late-stage nodular KS lesions [55,56]. Moreover, recent analysis of chromosomal abnormalities in KS shows increased numbers of recurrent (and sporadic) chromosomal alterations in nodular KS compared with the early cases [57].

We thus propose that the early steps of KSHV tumorigenesis involve activation of the DNA damage checkpoint. This enforces selective pressure for mutations abrogating the checkpoint, and provides an advantage for cells with defective DNA damage response components. KSHV v-cyclin–induced DNA damage, particularly the DSBs, may be one of the factors enhancing genomic instability and progression to KS. Whether it is the DNA damage checkpoint reported here that predominantly limits neoplastic transformation by KSHV remains to be determined.

## Materials and Methods

### Retrovirus production and transduction.

Amphotropic retroviruses were produced by transfection of Phoenix-Ampho retrovirus–producing cells (a kind gift from G. Nolan, Stanford University, Stanford, California, United States) with retroviral vectors using Lipofectamine 2000 reagent (Invitrogen, http://www.invitrogen.com/). After 48 h, viral supernatants were harvested and filtered through a 0.45-μm filter (Millipore, http://www.millipore.com/), and fresh media was added to Phoenix cells to produce more viral supernatant for a second round of virus production for 96 h.

Target cells were plated 1 d before, and spin-transduced using 8 μg/ml polybrene (Sigma, http://www.sigmaaldrich.com/) by centrifugation (2500 rpm; Heraeus Multifuge 3 S-R; Thermo Scientific, http://www.thermo.com/) for 30 min at room temperature. Cells were then returned to 37 °C, 5% CO_2_, and after 1 h of incubation viral supernatant was removed and replaced with fresh complete media. The transduction was highly efficient (80%–90%) as determined by flow cytometric analysis.

HDMECs (Promocell, http://www.promocell.com/) transduced with pWZLblast-hTERT were subjected to blasticidin selection (5 μg/ml) 2 d after transduction. Cells transduced with pBabe, 2FkpBabe, D3pBabe, or pBabepuro-H-RasV12 retrovirus were subjected 2 d after transduction to selection with 1 μg/ml puromycin, whereas cells transduced with pBabe-Hygro-p53Cter and pBabe-Hygro-SVLT retrovirus were subjected to selection with 200 μg/ml hygromycin B (Invitrogen). Retroviruses encoding the human papillomavirus oncogenes *E6* and *E7* (pLXSN-E6/E7) were produced as described earlier [58], and 2 d after transduction cells were subjected to selection with 700 μg/ml G418.

To construct CDK-silencing lentiviruses, shRNA-oligos to CDK2 (5′-GATCCGCACGTACGGAGTTGTGTATTCAAGAGATACACAACTCCGTACGTGCCCTTTTTTGGAAA-3′), CDK4 (5′-GATCCGCACTTACACCCGTGGTTGTTTCAAGAGAACAACCACGGGTGTAAGTGCCTTTTTTGGAAA-3′), CDK6 (5′-GATCCGAGTAGTGCATCGCGATCTTTCAAGAGAAGATCGCGATGCACTACTCGGTTTTTTGA-3′), and a noncoding, random sequence (Scramble; 5′-ATCCGTTCTCCGAACGTGTCACGTTTCAAGAGAACGTGACACGTTCGGAGAATTTTTTGGAAA-3′) were recombined from the pENTR-H1-BgH- vector (provided by the Biocentrum Helsinki SYSBIO initiative) by Gateway cloning technology (Invitrogen) into the lentiviral vector pDSL_hpUGIH (LGC Promochem, http://www.lgcpromochem.com/) according to the manufacturer's protocol. Viruses were produced by transfecting Invitrogen ViraPower viral packaging plasmids with pDSL_hpUGIH into 293FT cells with Lipofectamine 2000 reagent according to the manufacturer's protocol (Invitrogen). Lentiviral supernatants were collected 72 h after transfection, filtered through a 0.45-μm filter, and used for spin transduction of EA.hy926 cells as described above. Transduced cells were subjected for selection 72 h after transduction with 50 μg/ml hygromycin.

### rKSHV.219 production and infection.

Infectious recombinant KSHV virus (rKSHV.219) was produced from Vero cells latently infected with rKSHV.219 (a kind gift from J. Vieira, University of Washington, Seattle, Washington, United States), as described in [36]. hT-HDMECs, hT-HDMECs-p53CTer, hT-HDMECs-SVLT, or hT-HDMECs-E6/E7 were plated 1 d before, and were infected by the rKSHV.219 virus supernatant by spin-infection as described above for spin-transduction. At 2 d after infection, the cells were subjected to selection with 1 μg/ml puromycin.

## Supporting Information

Figure S1 DNA Damage Response in v-Cyclin–Expressing Primary HDMECs (A) Cells were transduced with v-cyclin–encoding retrovirus (KpBMN) and grown on coverslips for 3 d. Transduced cells were stained with antibodies against pS-ATM, pT-Chk2, and 53BP1 as indicated in the figure. The left panels show GFP expressed from the retrovirus. Scale bar = 20 μm.
(1.8 MB TIF)

Figure S2 Proliferation of the Late KSHV-ECs Is Accompanied by an Increase in LANA Signal (A) hT-HDMECs were infected with rKSHV.219 virus and grown for 6 d (early) or approximately 10 wk (late). Proliferation of these cells in relation to noninfected cells was determined by the MTT assay during a 5-d period.
(B) KSHV-ECs grown for 8 d after infection (early) or for 10 wk (late). Infected cells were labeled with anti-LANA antibodies (red) and Hoechst (blue). Quantitation for cells with more than 11 dots of LANA is indicated in the graph. Scale bar = 20 μm.
(589 KB TIF)

Figure S3 p53-Dependent Apoptosis Is Restrained in KSHV-Infected Endothelial Cells Late, post-crisis KSHV-ECs and their passage-matched, parental, noninfected ECs were treated with 7 μM Nutlin-3a. Cell viability was determined by trypan blue exclusion, and the percentage of dead cells was determined at 24, 48, and 96 h after the treatment. The values represent the percentage of apoptotic cells relative to the vehicle-treated control (i.e., percentage of apoptotic cells in vehicle-treated sample was subtracted from the percentage of apoptotic cells induced by Nutlin-3a).
(299 KB TIF)

Figure S4 DNA Damage Response Is Activated in Early-Stage KS Lesions (A) Paraffin-embedded sections of early-stage (Patch) and late-stage (Nodular) KS skin tumors were stained for pT-Chk2, and nuclei were counterstained with Hoechst 33342.
(B) Early-stage (Patch) and late-stage (Nodular) KS skin lesions were stained for γ-H2AX, and nuclei were counterstained with Hoechst 33342. Arrows indicate infiltrated red blood cells. The rightmost panels display magnifications of a marked area indicated by a yellow frame.
Images were captured at 20× and 40× magnification as indicated. Scale bars = 50 μM.
(6.1 MB TIF)

Figure S5 Specificity of the pT-Chk2 Staining Paraffin-embedded sections of early-stage KS skin tumors were stained with pT-Chk2 untreated (top panels) or pretreated with a peptide specific for the Thr68 phosphorylation site (bottom panels). The nuclei were counterstained with Hoechst 33342. Images were captured at 20× magnification. Scale bar = 50 μM.
(3.9 MB TIF)

Text S1 Supplemental Materials and Figure Legends
(219 KB DOC)

## Supporting Information

### Accession Numbers

The Genbank (http://www.ncbi.nlm.nih.gov/Genbank/) accession numbers for the genes and gene products discussed in this paper are *ATM* (NM 000051), *ATR* (NM 001184), *Cdc6* (NM 001254), *Cdc25A* (NM 001789), *CDK1* (NM 033379), *CDK2* (NM 001798), *CDK4* (NM 000075), *CDK6* (NM 001259), *Chk2* (NM 007194), *cyclin B1* (NM 031966), *cyclin D3* (NM 001760), *cyclin E* (NM 001238), *E2F1* (NM 005225), *E1A* (AY147066), *histone H2* (XM 636495), *histone H3* (NM 003493), *hTERT* (NM 198253), human papillomavirus *E6* (EF424414), human papillomavirus *E7* (AF478148), *K-Ras* (M54968), *LANA* (AF305694), *Myc* (NM 012333), *Orc 1* (U40152), *pRb* (AF109873), *p21Cip1* (NM 000389), *p27Kip1* (NM 004064), *p53* (NM 000546), SV40 *large T antigen* (AF168998), *v-cyclin* (U79416), *vFLIP* (U90534), and *53BP1* (NM 005657).

## Abbreviations

53BP1: p53-binding protein
DSB: double-strand break
EC: endothelial cell
HDMEC: human dermal microvascular EC
hTERT: human telomerase
KS: Kaposi sarcoma
KSHV: Kaposi sarcoma herpesvirus
PEL: primary effusion lymphoma
shRNA: small hairpin RNA
