# Supplementary figures and images for: Viral Oncogene–Induced DNA Damage Response Is Activated in Kaposi Sarcoma Tumorigenesis
Source: PLoS Pathog. 2007 Sep 28;3(9):e140. doi: 10.1371/journal.ppat.0030140 (PMC1994968; doi:10.1371/journal.ppat.0030140)

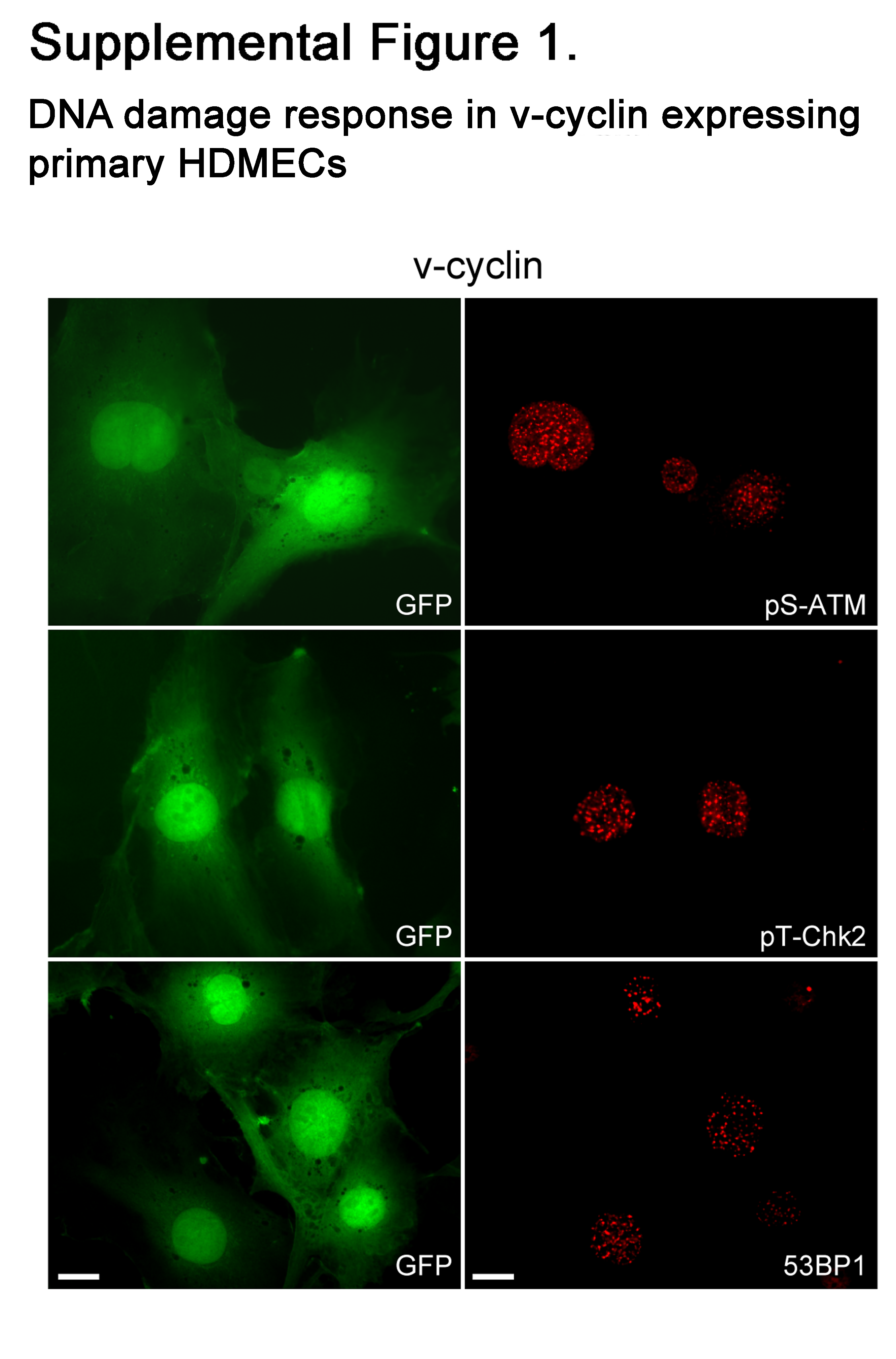

Supplement: Figure S1 — (A) Cells were transduced with v-cyclin–encoding retrovirus (KpBMN) and grown on coverslips for 3 d. Transduced cells were stained with antibodies against pS-ATM, pT-Chk2, and 53BP1 as indicated in the figure. The left panels show GFP expressed from the retrovirus. Scale bar = 20 μm. (1.8 MB TIF) [file ppat.0030140.sg001.tif]

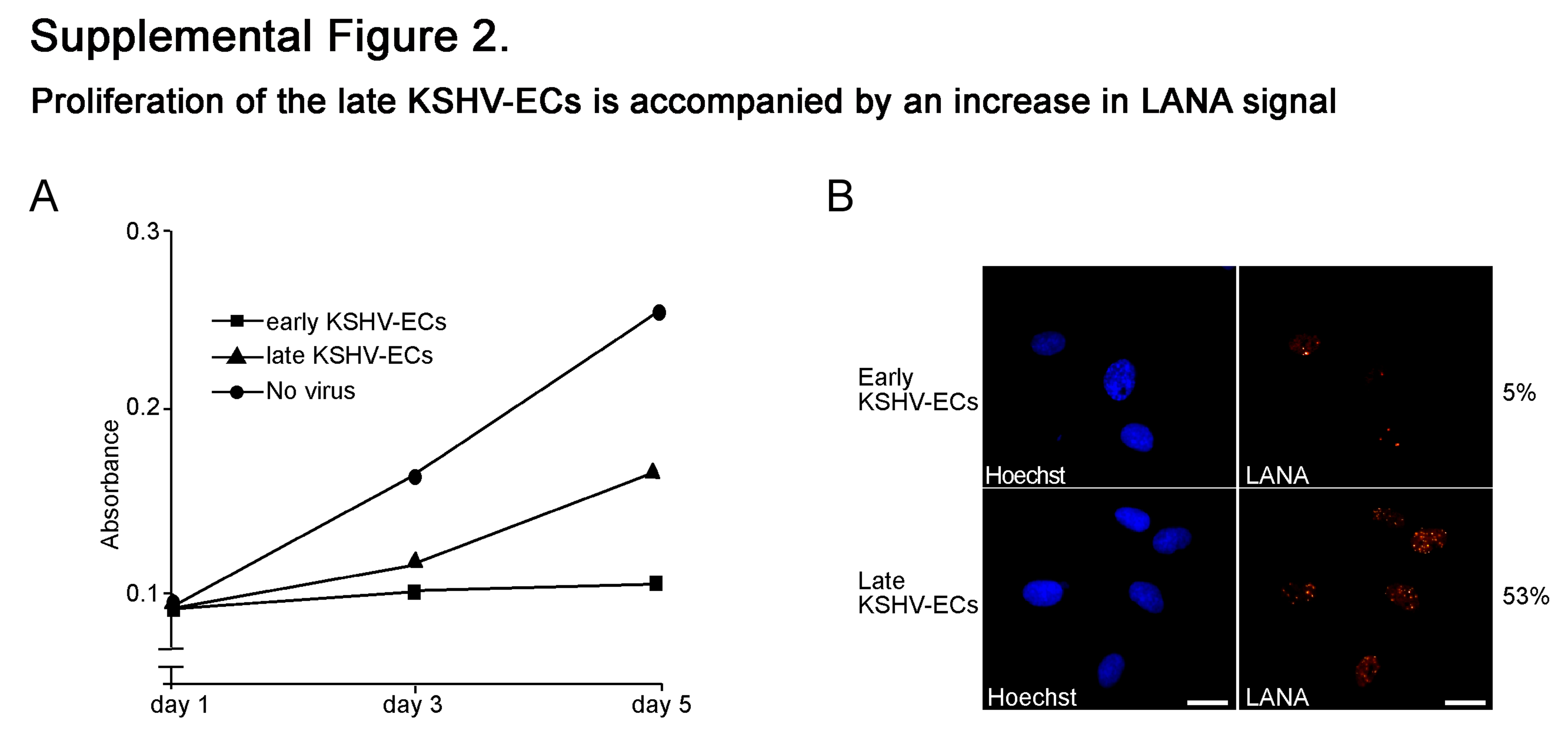

Supplement: Figure S2 — (A) hT-HDMECs were infected with rKSHV.219 virus and grown for 6 d (early) or approximately 10 wk (late). Proliferation of these cells in relation to noninfected cells was determined by the MTT assay during a 5-d period. (B) KSHV-ECs grown for 8 d after infection (early) or for 10 wk (late). Infected cells were labeled with anti-LANA antibodies (red) and Hoechst (blue). Quantitation for cells with more than 11 dots of LANA is indicated in the graph. Scale bar = 20 μm. (589 KB TIF) [file ppat.0030140.sg002.tif]

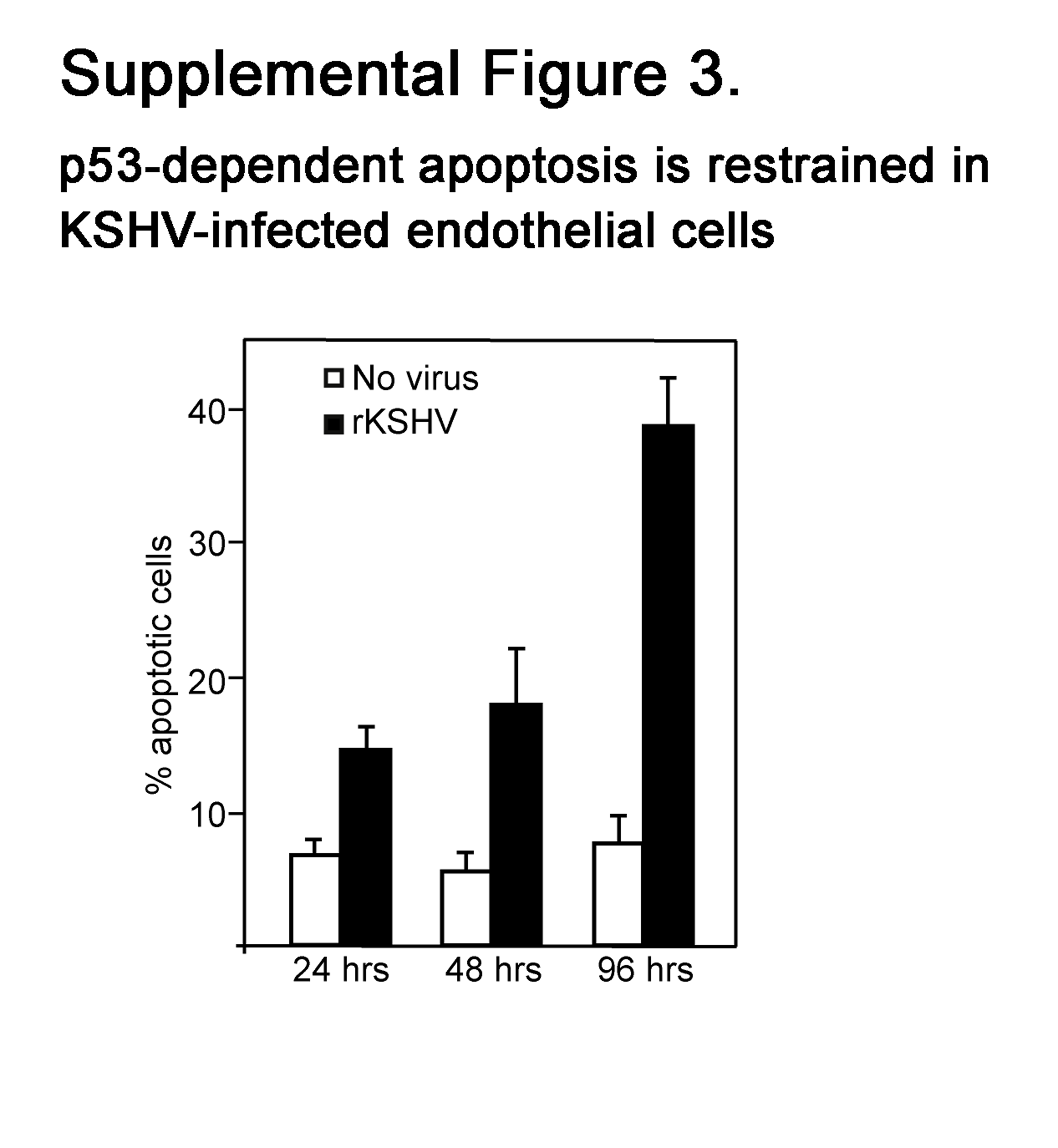

Supplement: Figure S3 — Late, post-crisis KSHV-ECs and their passage-matched, parental, noninfected ECs were treated with 7 μM Nutlin-3a. Cell viability was determined by trypan blue exclusion, and the percentage of dead cells was determined at 24, 48, and 96 h after the treatment. The values represent the percentage of apoptotic cells relative to the vehicle-treated control (i.e., percentage of apoptotic cells in vehicle-treated sample was subtracted from the percentage of apoptotic cells induced by Nutlin-3a). (299 KB TIF) [file ppat.0030140.sg003.tif]

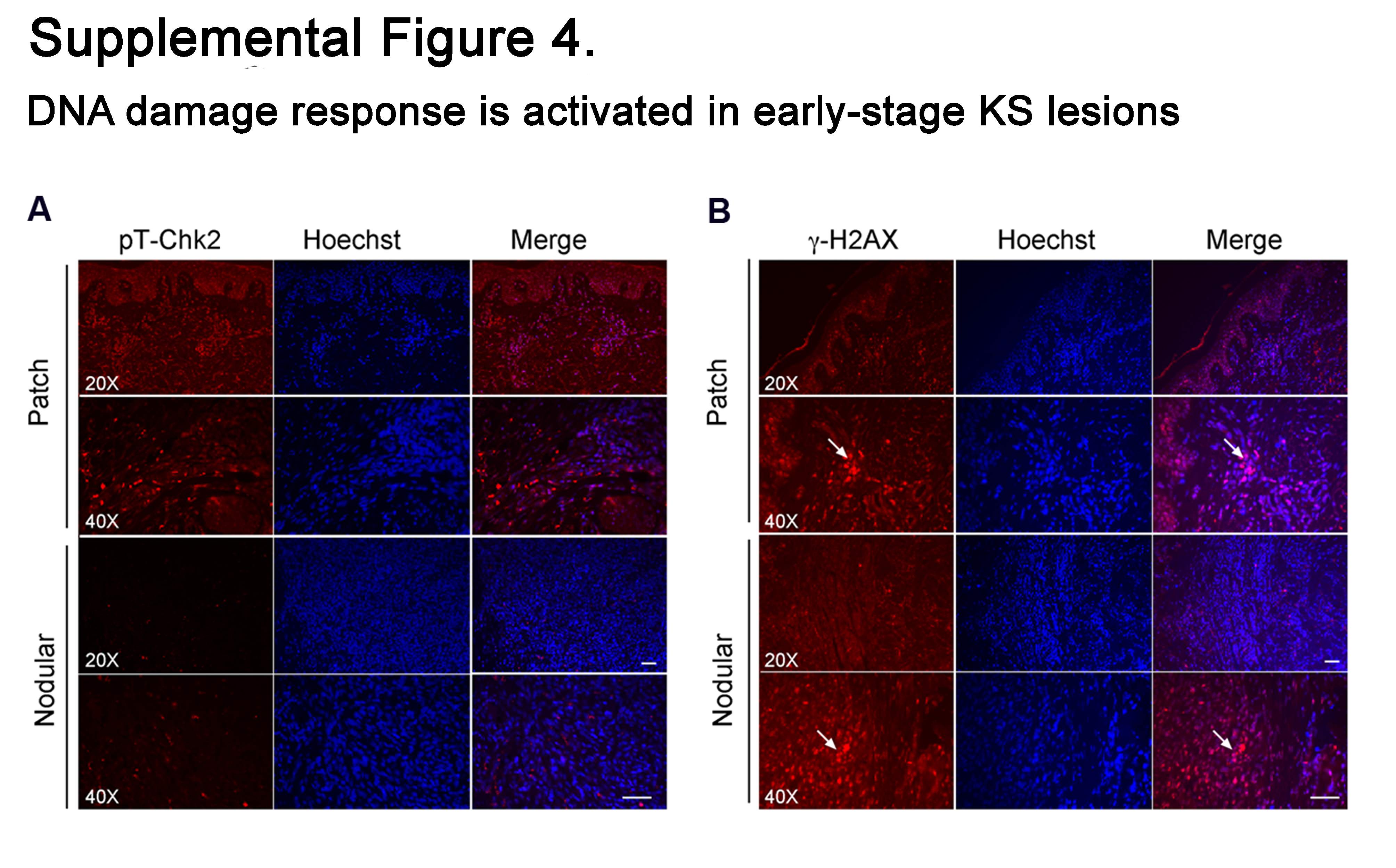

Supplement: Figure S4 — (A) Paraffin-embedded sections of early-stage (Patch) and late-stage (Nodular) KS skin tumors were stained for pT-Chk2, and nuclei were counterstained with Hoechst 33342. (B) Early-stage (Patch) and late-stage (Nodular) KS skin lesions were stained for γ-H2AX, and nuclei were counterstained with Hoechst 33342. Arrows indicate infiltrated red blood cells. The rightmost panels display magnifications of a marked area indicated by a yellow frame. Images were captured at 20× and 40× magnification as indicated. Scale bars = 50 μM. (6.1 MB TIF) [file ppat.0030140.sg004.tif]

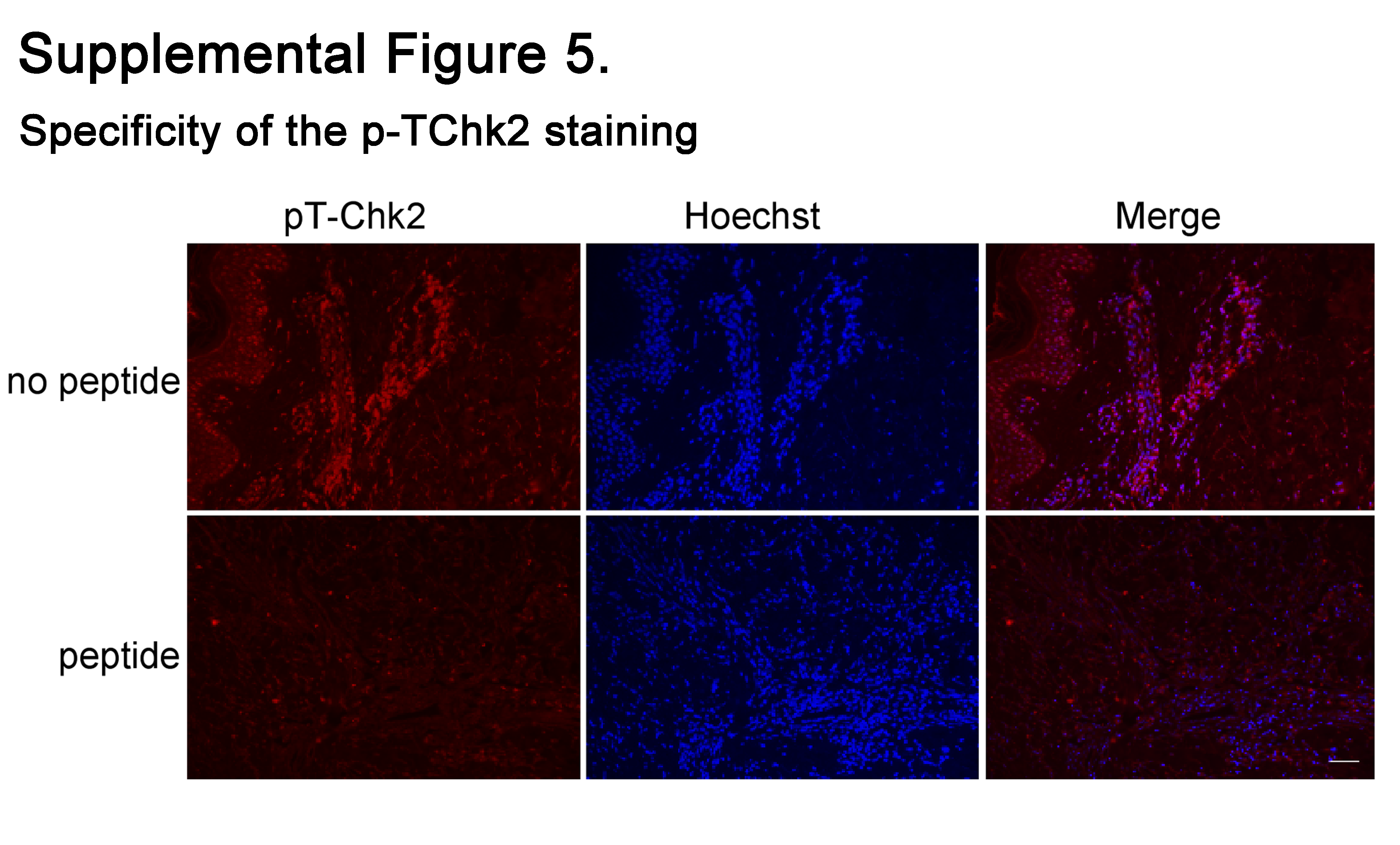

Supplement: Figure S5 — Paraffin-embedded sections of early-stage KS skin tumors were stained with pT-Chk2 untreated (top panels) or pretreated with a peptide specific for the Thr68 phosphorylation site (bottom panels). The nuclei were counterstained with Hoechst 33342. Images were captured at 20× magnification. Scale bar = 50 μM. (3.9 MB TIF) [file ppat.0030140.sg005.tif]
