# Supplementary material for: Viral Oncogene–Induced DNA Damage Response Is Activated in Kaposi Sarcoma Tumorigenesis
Source: PLoS Pathog. 2007 Sep 28;3(9):e140. doi: 10.1371/journal.ppat.0030140 (PMC1994968; doi:10.1371/journal.ppat.0030140)
Supplement: Text S1 — Supplemental Materials and Figure Legends (219 KB DOC) [file ppat.0030140.sd001.doc]

**Supplemental Figure legends**

**Supplemental Figure 1.** DNA damage response in v-cyclin expressing primary HDMECs. (A) Cells were transduced with v-cyclin-encoding retrovirus (KpBMN) and grown on coverslips for 3 days. Transduced cells were stained with antibodies against pS-ATM, pT-Chk2 and 53BP1 as indicated in the figure. The left panels show GFP expressed from the retrovirus. Scale bar = 20µm.

**Supplemental Figure 2.** Proliferation of the late KSHV-ECs is accompanied by an increase in LANA signal. (A) hT-HDMECs were infected with rKSHV.219 virus and grown for 6 days (early) or approximately 10 weeks (late). Proliferation of these cells in relation to non-infected cells was determined by the MTT assay during a 5-day period. (B) KSHV-ECs grown for 8 days post infection (early) or for 10 weeks (late). Infected cells were labeled with anti-LANA antibodies (red) and Hoechst (blue). Quantitation for cells with more than 11 dots of LANA is indicated in the graph. Scale bar = 20µm.

**Supplemental Figure 3.** p53-dependent apoptosis is restrained in KSHV-infected endothelial cells. Late, post-crisis KSHV-ECs, and their passage-matched parental, non-infected ECs were treated with 7µM Nutlin-3a. Cell viability was determined by trypan blue exclusion, and the percentage of dead cells was determined at 24, 48 and 96 h after the treatment. The values represent the percentage of apoptotic cells relative to the vehicle-treated control (i.e. % of apoptotic cells in vehicle-treated sample was subtracted from % of apoptotic cells induced by Nutlin-3a).

**Supplemental Figure 4.** DNA damage response is activated in early-stage KS lesions. (A) Paraffin-embedded sections of early stage (Patch) and late stage (Nodular) KS skin tumors were stained for pT-Chk2 and nuclei were counterstained with Hoechst 33342. (B) Early (Patch) and late (Nodular) stage KS skin lesions were stained for γ-H2AX and nuclei were counterstained with Hoechst 33342. Arrows indicate infiltrated red blood cells**.** The rightmost panels display magnifications of a marked area indicated by a yellow frame. Images were captured at 20X and 40X magnification as indicated. Scale bars = 50µM.

**Supplemental Figure 5.** Specificity of the pT-Chk2 staining. Paraffin-embedded sections of early stage KS skin tumors were stained with pT-Chk2 untreated (top panels) or pretreated with a peptide specific for the Thr68 phosphorylation site (bottom panels). The nuclei were counterstained with Hoechst 33342. Images were captured at 20x magnification. Scale bar = 50µM.

# Supplementary Methods

**Cell culture**

HDMECs were maintained in the Endothelial cell growth medium (Promocell, Heidelberg, Germany) containing 10 ng/ml epidermal growth factor, 0.4% endothelial cell growth supplement/heparin, 5% (wt/vol) Fetal Calf Serum (FCS), 1 μg/ml hydrocortisone, 50 μg/ml gentamicin and 0.05 μg/ml amphotericin (Promocell). The EA.hy 926 cell line was derived by fusing human umbilical vein endothelial cells with the permanent human cell line A549 [1], and it retains wild-type p53 as well as expression of several endothelial cell markers and properties [2]. EA.hy 926 cells, PA317 packaging cell (expressing papillomavirus E6/E7 oncogenes; a gift from K. Wartiovaara, University of Helsinki, Helsinki, Finland) lines, and rKSHV.219-infected Vero cells [3] were routinely cultured in Dulbecco´s modified Eagle´s medium (DMEM), supplemented with 10% FCS, 2 mM glutamine, 100 μg/ml streptomycin, and 100 U/ml penicillin. For the PA317 packaging cell line, an additional 700 μg/ml of G418 (Roche, Basel, Switzerland) was added to the medium, and for the rKSHV.219-Vero cells 5 μg/ml of puromycin (Sigma, St Louis, MO). Phoenix-Ampho retrovirus packaging cells (a gift from G. Nolan, Stanford University, Stanford, CA) were maintained in the same supplemented DMEM as described above with additional 4.5 g glucose. All cells were routinely cultured in a humidified 5% CO_2_ atmosphere at +37°C.

**Antibodies and reagents**

The antibodies used were the following: α-pS-ATM (Ser1981), α-pS-p53 (Ser15), α-pT-Chk2 (Thr68) all from Cell Signaling Technology, Inc (Danvers, MA); anti-BrdU (clone Bu20a) from DakoCytomation (Glostrup, Denmark); α-CDK6 (C-21), α-CDK4 (C-22), α-CDK2 (M2), α-cyclin B1 (GNS-1), and α-p21 (C-19) from Santa Cruz Biotechnology (Santa Cruz, CA); α-LANA (ORF73) from Advanced Biotechnologies (Columbia, MD); anti-γ-tubulin (GTU-88) and α-Flag (F7425) from Sigma; α-cdc6 (DCS-180) and anti-γ-H2AX (JBW301) from Upstate Biotechnology (Lake Placid, NY); α-53BP1 from Novus Biologicals, Inc (Littleton, CO); anti-β-tubulin (5H1) from BD Biosciences (Hercules, CA); and α-GFP (TP401) from Torrey Pines Biolabs, Inc (Houston, TX). Mouse monoclonal antibody recognizing p53 (p53 DO-1) was prepared by M. Laiho. Alexa 488- and 594-conjugated secondary antibodies were purchased from Molecular Probes (Eugene, OR); FITC-conjugated anti-Rat and horseradish peroxidase (HRP)-conjugated secondary antibodies were from Chemicon (Temecula, CA). The ATM inhibitor KU-55933 (K4014), dimethyl Sulfoxide (DMSO), trypan blue (0.4%), wortmannin, and Bisbenzimide Hoechst 33342 from Sigma-Aldrich (St Louis, MO), and caffeine from Calbiochem (Darmstadt, Germany).

.

**Plasmids**

The retroviral vector pWZLblast-hTERT was obtained from the Biomedicum Helsinki Virus core (<http://research.med.helsinki.fi/corefacilities/bvc/>), pBabepuro (pBabe), double Flag-tagged v-cyclin subcloned into pBabepuro (2FkpBabe), bicistronic pBMNIresEGFP (pBMN), double Flag-tagged v-cyclin subcloned into pBMN (KpBMN) and double Flag-tagged cyclin D3 in pBabe (D3pBabe) were gifts from E. Verschuren (Standford University, Stanford, CA) [4] and D. Mann (Imperial College London, London, United Kingdom). The pBabe-Hygro-p53CTer vector expressing the carboxy-terminal amino acid residues 302-390 of murine p53 were gifts from J. Klefström, (University of Helsinki, Helsinki, Finland) [5]. The pBabe-Hygro-SVLT retroviral vector was a gift from L. Raptis (Queen´s University Kingston, Ontario, Canada). The pBabePuro-H-RasV12 was a gift from S. Lowe, (Cold Spring Harbor Laboratory, Cold Spring Harbor, NY). The lentiviral vector pDSL_hpUGIH for shRNA expression was obtained from Cell signaling Technology, Inc.

**Proliferation assay**

2000 cells were seeded in a 96-well plate. At indicated times, proliferation was determined by using a cell proliferation kit (MTT) (Roche, Basel, Switzerland) according to manufacturer´s protocol. The assay measures metabolic activity of the cells as an indication of proliferation.

**Flow cytometric analysis**

Semi-confluent cell cultures were labeled with 15 μM 5-BrdU (Sigma) for 2 hr. Cells were fixed with ice-cold 70% ethanol and treated with 0.7 N HCl for 30 min at RT to denature DNA. Cells were stained with anti-BrdU-antibody, and Alexa 488-conjugated secondary antibody, followed by incubation with 30 μg/ml propidium iodide and 30 μg/ml RNase A (both from Sigma) at +37 °C for 30 min. All analyses were performed with BD LSR flow cytometer (BD Biosciences, Franklin Lakes, New Jersey) and cell cycle analysis was performed with ModFit LT.

**Senescence assay**

hTERT-HDMECs transduced with pBabe, 2FKpBabe. or pBABE-H-RasV12 were selected with puromycin for 5 days, and fixed in 2% formaldehyde, 0.2% glutaraldehyde in PBS, for 10 min at RT. After fixation, cells were washed and the SA-β-gal stain solution (7.4 mM citric acid-25 mM sodium phosphate, pH 6.0; 5 mM potassium ferrocyanate; 5 mM potassium ferricyanide; 150 mM sodium chloride, 2 mM magnesium chloride; 1mg/ml X-gal (5-bromo-4-chloro-3-indolyl β-D-galactosidase) [6] was added. The cells were incubated at 37 °C for 16 hr, and analyzed with phase contrast microscopy.

**Immunoblotting**

Whole cell extracts were prepared essentially as previously described [7]. Alternatively, for detection of DNA damage markers, cells were lysed in urea-Tris buffer (UTB) (9 M urea, 75 mM Tris-HCL, pH 7.5, 0.15 M 2-mercaptoethanol) and sonicated briefly. Protein concentrations were determined by BioRad DC assay (Bio-Rad, Hercules, CA) according to the manufacturer´s protocol. Immunoblotting was performed as described in [7].

**Indirect immunofluorescence**

For centrosome labeling, cells were either fixed in ice-cold methanol (for the 2FKpBabe or pBabe transduced cells) for 10 min at –20°C and or in the PHEMO fix (for the KpBMN or pBMN transduced cells; 2 x PHEMO-fix: 7.4 % paraformaldehyde (PFA), 0.1% glutaraldehyde, 1% TX-100), and processed as described in [8]. Coverslips were blocked with 5% goat serum in PBS for 30 min, followed by staining for with anti-γ-tubulin, and with a fluorochrome-conjugated secondary antibody. For pS-ATM, pT-Chk2, 53BP1, cyclin B1, and LANA labeling, cells were fixed on coverslips with 4% PFA for 15 min and permeabilized by 0.1 % TX-100 for 5 min. Blocking and staining was performed as described in [7]. For the double-labelings (γ-H2AX-Flag or γ-H2AX-GFP), the cells were fixed on coverslips with 4% PFA for 20 min, washed with PBS, and permeabilized with ice-cold 70% ethanol for 5 min at –20°C. After washing, cells were blocked with 8% BSA in PBS for 1 h, and processed as previously described [7]. DNA was counterstained with Hoechst 33342 (0.5 μg/ml) for 5 minutes, and coverslips were mounted in MOWIOL (Calbiochem, La Jolla, USA), followed by microscopic analysis with a Zeiss Axioplan 2 fluorescent microscope (Carl Zeiss). Images were acquired with a Zeiss Axiocam HRc, using Zeiss AxioVision and Adobe Photoshop 7.0 (Adobe) software.

**In vitro kinase assay**

For measurements of cyclin B1-associated kinase activity, hT-HDMECs were transduced with pBMN or KpBMN retroviruses essentially as described above for retroviral transduction. As a positive control, the cells were treated for 20 hr with 75 ng/ml nocodazole (Sigma). Cells were lysed into ELB lysis buffer [7] supplemented with 25 mM β-glycerophosphate, and 300 μg of lysate was incubated for 2 hr at +4°C with the anti-cyclin B1 antibody (Santa Cruz, CA) antibody. In vitro kinase assay was performed as described earlier [9], with the exception of using Gammabind Sepharose beads (Sigma) to couple the immunocomplexes. Phosphorylated proteins were analyzed by SDS-PAGE and autoradiography.

**Immunohistochemistry**

Paraffin-embedded tissue samples were deparaffinized, rehydrated, and the antigens retrieved in citrate buffer (pH 6.0, Antigen Retrieval Solution, DakoCytomation) using a microwave oven (780W, at least 6 min for LANA and pT-Chk2, 800W, 18 min for γ-H2AX and 53BP1). The slides were blocked in 5% normal goat serum for LANA, 5% normal swine serum for pT-Chk2, and 5% milk/0.25% Triton-X-100 in PBS supplied with 3% goat normal serum for γ-H2AX and 53BP1 for 30 min. The primary antibodies were diluted in the blocking solution and incubated overnight at +4°C. After incubating the sections with appropriate fluorochrome-conjugated secondary antibodies for 30 – 40 min, they were counterstained with Hoechst (1ug/ml) or DAPI and mounted. The slides were analysed with a Zeiss Axioplan 2 fluorescent microscope (Carl Zeiss). Images were acquired with a Zeiss Axiocam HRc, using Zeiss AxioVision and Adobe Photoshop 7.0 (Adobe) software.

**Imaging equipment and settings**

**Microscope:** Zeiss Axioplan 2 upright epifluorescence microscopes

**Objective lenses:**

Zeiss Plan-Neofluar, 20X, NA 0.50, (model 1004-989)

Zeiss Plan-Neofluar, 40X, NA 0.75, (model 440351)

Zeiss Plan-Neofluar, 63X, NA 1.25, (model 440460)

Zeiss Plan-Neofluar, 100X, NA 1.30, (model 1031-171)

**Filter sets:**

DAPI; ex filter spectrum D360/40, (ex filter cat.no: 39869), em filter spectrum

D460/50, (em filter cat no: 39194).

FITC; ex filter spectrum HQ480/40, (ex filter cat.no: 39552), em filter spectrum

HQ535/50, (em filter cat no: 38074).

Texas Red; ex filter spectrum HQ560/55, (ex filter cat.no: 36363), em filter spectrum

HQ645/75, (em filter cat no: 35279).

TRITC; ex filter spectrum HQ545/30, (ex filter cat.no: 36835), em filter spectrum

HQ610/75, (em filter cat no: 36760).

**Camera:** AxioCam HRc, color 3x14 bit

**Acquisition Software:**

Zeiss Axiovision 4.4

Adobe Photoshop 7.0 (Adobe, San Jose, CA) software

AutoQuant (version X1.4.0): 2D blind deconvolution

**Channels:**

DAPI

Excitation wavelength: 352

Emission wavelength: 461

FITC

Excitation wavelength: 494

Emission wavelength: 518

TRITC

Excitation wavelength: 541

Emission wavelength: 572

Alexa 594:

Excitation wavelength: 590

Emission wavelength: 617

**Figure 2A:**

Objective magnification 63xx: scale factor for x and y= 0.17 (µm/pixel).

Pixel type: 48 bit RGB color

Acquisition bit depth: 42

Axiocham resolution: 1300x1030 standard color

Channel: FITC

Exposure time:

GFP: 650ms

FLAG: 100 ms

Channel: Alexa 594

Exposure time:

p-ATM (Ser 1981): 100ms

γ-H2AX: 150ms

p-Chk2 (Thr68): 150ms

53BP1: 140ms

**Figure 3A:**

Objective magnification 100xx: scale factor for x and y= 0.11 (µm/pixel).

Pixel type: 24 bit RGB color

Acquisition bit depth: 42

Axiocham resolution: 1300x1030 standard color

Channel: DAPI

Exposure time: Hoechst: 20ms

Channel: Alexa 594

Exposure time: γ-tubulin: 251ms

**Figure 3C:**

Objective magnification 40x: scale factor for x and y= 0.267737 (µm/pixel).

Pixel type: 24 bit RGB color

Acquisition bit depth: 42

Axiocham resolution: 1300x1030 standard color

Channel: FITC

Exposure time:

GFP: 600ms

Channel: DAPI

Exposure time:

Hoechst: 130ms

**Figure 3E:**

Objective magnification 63xx: scale factor for x and y= 0.17 (µm/pixel).

Pixel type: 24 bit RGB color

Acquisition bit depth: 42

Axiocham resolution: 1300x1030 standard color

Channel: DAPI

Exposure time: Hoechst: 176ms

Channel: Alexa 594

Exposure time: cyclin B1: 682ms

**Figure 4A:**

Objective magnification 63xx: scale factor for x and y= 0.17 (µm/pixel).

Pixel type: 24 bit RGB color

Acquisition bit depth: 42

Axiocham resolution: 1300x1030 standard color

Channel: DAPI

Exposure time: Hoechst: 26ms

Channel: Alexa 594

Exposure time:

p-ATM (Ser 1981): 130ms

p-Chk2 (Thr68): 270ms

**Figure 5B:**

*Upper panel*

Objective magnification 40xx: scale factor for x and y= 0.267737 (µm/pixel).

Pixel type: 24 bit RGB color

Acquisition bit depth: 42

Axiocham resolution: 1300x1030 standard color

Channel: DAPI

Exposure time: Hoechst: 124 ms

Channel: FITC

Exposure time: GFP: 718 ms

*Lower panel*

Objective magnification 100xx: scale factor for x and y= 0.11 (µm/pixel).

Pixel type: 24 bit RGB color

Acquisition bit depth: 42

Axiocham resolution: 1300x1030 standard color

Channel: DAPI

Exposure time: Hoechst: 31ms

Channel: Alexa 594

Exposure time: γ-tubulin: 149ms

**Figure 5D:**

Objective magnification 63xx: scale factor for x and y= 0.17 (µm/pixel).

Pixel type: 24 bit RGB color

Acquisition bit depth: 42

Axiocham resolution: 1300x1030 standard color

Channel: Alexa 594

Exposure time: 53BP1: 300ms

Channel: DAPI

Exposure time: Hoechst: 52ms

**Figure 6A:**

*Patch*

Objective magnification 40xx: scale factor for x and y= 0.267737 (µm/pixel).

Pixel type: 48 bit RGB color

Acquisition bit depth: 42

Axiocham resolution: 1300x1030 standard color

Channel: TRITC

Exposure time: p-Chk2 (Thr68): 1119ms

*Nodular*

Objective magnification 40xx: scale factor for x and y= 0.267737 (µm/pixel).

Pixel type: 48 bit RGB color

Acquisition bit depth: 42

Axiocham resolution: 1300x1030 standard color

Channel: TRITC

Exposure time: p-Chk2 (Thr68): 999ms

**Figure 6B:**

Objective magnification 63xx: scale factor for x and y= 0.17 (µm/pixel).

Pixel type: 48 bit RGB color

Acquisition bit depth: 42

Axiocham resolution: 1300x1030 standard color

Channel: TRITC

Exposure time:

LANA: 984ms

p-Chk2 (Thr68): 307ms

**Figure 6C:**

*Patch*

Objective magnification 40xx: scale factor for x and y= 0.267737 (µm/pixel).

Pixel type: 48 bit RGB color

Acquisition bit depth: 42

Axiocham resolution: 1300x1030 standard color

Channel: Alexa 594

Exposure time: γ-H2AX: 999ms

*Nodular*

Objective magnification 40xx: scale factor for x and y= 0.267737 (µm/pixel).

Pixel type: 48 bit RGB color

Acquisition bit depth: 42

Axiocham resolution: 1300x1030 standard color

Channel: Alexa 594

Exposure time: γ-H2AX: 1191ms

**Figure 6D:**

Objective magnification 63xx: scale factor for x and y= 0.17 (µm/pixel).

Pixel type: 48 bit RGB color

Acquisition bit depth: 42

Axiocham resolution: 1300x1030 standard color

Channel: Alexa 594

Exposure time:

53BP1 (upper panel): 655ms

53BP1 (lower panel): 349ms

**Supplemental Figure 1:**

Objective magnification 63xx: scale factor for x and y= 0.17 (µm/pixel).

Pixel type: 24 bit RGB color

Acquisition bit depth: 42

Axiocham resolution: 1300x1030 standard color

Channel: FITC

Exposure time: GFP: 650ms

Channel: Alexa 594

Exposure time:

p-ATM (Ser 1981): 76ms

p-Chk2 (Thr68): 170ms

53BP1: 156ms

**Supplemental Figure 2B:**

Objective magnification: 63xx: scale factor for x and y= 0.17 (µm/pixel).

Pixel type: 24 bit RGB color

Acquisition bit depth: 42

Axiocham resolution: 1300x1030 standard color

Channel: DAPI

Exposure time: Hoechst: 191ms

Channel: TRITC

Exposure time: LANA: 394ms

**Supplemental Figure 4A:**

*Patch*

Objective magnification 40xx: scale factor for x and y= 0.267737 (µm/pixel).

Pixel type: 48 bit RGB color

Acquisition bit depth: 42

Axiocham resolution: 1300x1030 standard color

Channel: TRITC

Exposure time: p-Chk2 (Thr68): 1119ms

Objective magnification 20xx: scale factor for x and y= 0.52 (µm/pixel).

Pixel type: 48 bit RGB color

Acquisition bit depth: 42

Axiocham resolution: 1300x1030 standard color

Channel: TRITC

Exposure time: p-Chk2 (Thr68): 1759ms

*Nodular*

Objective magnification 40xx: scale factor for x and y= 0.267737 (µm/pixel).

Pixel type: 48 bit RGB color

Acquisition bit depth: 42

Axiocham resolution: 1300x1030 standard color

Channel: TRITC

Exposure time: p-Chk2 (Thr68): 999ms

Objective magnification 20xx: scale factor for x and y= 0.52 (µm/pixel).

Pixel type: 48 bit RGB color

Acquisition bit depth: 42

Axiocham resolution: 1300x1030 standard color

Channel: TRITC

Exposure time: p-Chk2 (Thr68): 1299ms

**Supplemental Figure 4B:**

*Patch*

Objective magnification 40xx: scale factor for x and y= 0.267737 (µm/pixel).

Pixel type: 48 bit RGB color

Acquisition bit depth: 42

Axiocham resolution: 1300x1030 standard color

Channel: Alexa 594

Exposure time: γ-H2AX: 999ms

Objective magnification 20xx: scale factor for x and y= 0.52 (µm/pixel).

Pixel type: 48 bit RGB color

Acquisition bit depth: 42

Axiocham resolution: 1300x1030 standard color

Channel: Alexa 594

Exposure time: γ-H2AX: 1189ms

*Nodular*

Objective magnification 40xx: scale factor for x and y= 0.267737 (µm/pixel).

Pixel type: 48 bit RGB color

Acquisition bit depth: 42

Axiocham resolution: 1300x1030 standard color

Channel: Alexa 594

Exposure time: γ-H2AX: 1191ms

Objective magnification 20xx: scale factor for x and y= 0.52 (µm/pixel).

Pixel type: 48 bit RGB color

Acquisition bit depth: 42

Axiocham resolution: 1300x1030 standard color

Channel: TRITC

Exposure time: γ-H2AX: 1188ms

**Supplemental Figure 5:**

Objective magnification 20xx: scale factor for x and y= 0.52 (µm/pixel).

Pixel type: 48 bit RGB color

Acquisition bit depth: 42

Axiocham resolution: 1300x1030 standard color

Channel: TRITC

Exposure time:

p-Chk2 (Thr68) (Peptide): 1189ms

p-Chk2 (Thr68) (No peptide): 1189ms

## REFERENCES

1. Edgell, C.J., C.C. McDonald, and J.B. Graham, (1983). Permanent cell line expressing human factor VIII-related antigen established by hybridization*.* Proc Natl Acad Sci U S A, 80(12): p. 3734-7.

2. Rieber, A.J., et al., (1993). Extent of differentiated gene expression in the human endothelium-derived EA.hy926 cell line*.* Thromb Haemost, 69(5): p. 476-80.

3. Vieira, J. and P.M. O'Hearn, (2004). Use of the red fluorescent protein as a marker of Kaposi's sarcoma-associated herpesvirus lytic gene expression*.* Virology, 325(2): p. 225-40.

4. Verschuren, E.W., et al., (2002). The oncogenic potential of Kaposi's sarcoma-associated herpesvirus cyclin is exposed by p53 loss in vitro and in vivo*.* Cancer Cell, 2(3): p. 229-41.

5. Klefstrom, J., et al., (1997). Induction of TNF-sensitive cellular phenotype by c-Myc involves p53 and impaired NF-kappaB activation*.* Embo J, 16(24): p. 7382-92.

6. Palmero, I. and M. Serrano, (2001). Induction of senescence by oncogenic Ras*.* Methods Enzymol, 333: p. 247-56.

7. Sarek, G., A. Jarviluoma, and P.M. Ojala, (2006). KSHV viral cyclin inactivates p27KIP1 through Ser10 and Thr187 phosphorylation in proliferating primary effusion lymphomas*.* Blood, 107(2): p. 725-32.

8. Dohner, K., et al., (2002). Function of dynein and dynactin in herpes simplex virus capsid transport*.* Mol Biol Cell, 13(8): p. 2795-809.

9. Jarviluoma, A., et al., (2004). KSHV viral cyclin binds to p27KIP1 in primary effusion lymphomas*.* Blood, 104(10): p. 3349-54.
